# Supplementary material for: Propagation pathways of Indo-Pacific rainfall extremes are modulated by Pacific sea surface temperatures
Source: Nat Commun. 2023 Sep 15;14:5708. doi: 10.1038/s41467-023-41400-9 (PMC10504381; doi:10.1038/s41467-023-41400-9)
Supplement: Supplementary file 1 — Supplementary Information [file 41467_2023_41400_MOESM1_ESM.pdf]

Supporting Material to Propagation pathways of Indo-Pacific rainfall extremes are modulated by Pacific sea surface temperatures

Felix M. Strnad[1,*], Jakob Schloer[1], Ruth Geen[2], Niklas Boers[3,4,5], and Bedartha Goswami[1,*]

[1]Cluster of Excellence Machine Learning: New Perspectives for Science, University of Tübingen, Tübingen, Germany
[2]School of Geography, Earth and Environmental Sciences, University of Birmingham, Birmingham, UK
[3]School of Engineering & Design, Earth System Modelling, Technical University Munich, Munich, Germany
[4]Potsdam Institute for Climate Impact Research, Potsdam, Germany
[5]Department of Mathematics and Global Systems Institute, University of Exeter, Exeter, UK
[*]Corr. authors: felix.strnad@uni-tuebingen.de

ABSTRACT

Contents of this file: Text Supplementary Note 1 to Supplementary Note 14; Figures S1 to S41

Introduction

In this Supplementary Material to our article, we describe in detail the definition of Extreme Rainfall Events (EREs) in the Indo-Asia Pacific domain (Text Supplementary Note 1, Fig. S1). The schematic of the Event Synchronization approach is discussed in Sec. Supplementary Note 2 and Fig. S2. We show one schematic to exemplify the climate network approach (Fig. S3). A detailed analysis of the regions of synchronous EREs, detected by a community detection approach is given in Supplementary Note 3. Here, we discuss as well the synchronous rainfall index estimation per community (Fig. S4,S5) as well as its lead-lag behavior (Fig. S6). We also analyzed the synchronous rainfall indices with respect to their periodicity (Fig. S7). Also a Hovmöller diagram for initiation time points in the equatorial Indian Ocean (EIO) are shown in Fig. S8. Intra-annual distribution of occurrence of synchronous EREs is explored in section Supplementary Note 4 (Fig. S9, S10). In Sec. Supplementary Note 5, we show one example for the canonical BSISO propagation (Fig. S11, S12), as well as the spatially resolved propagation of the BSISO for the three discovered BSISO propagation modes (Fig. S13, Fig. S14, Fig. S15). The corresponding propagation of EREs is shown for the three modes in Fig. S16. The robustness of our results with respect to the chosen BSISO index is shown in section Supplementary Note 6, Fig. S17. We demonstrate in section Sec. Supplementary Note 7 that the organization of EREs through the BSISO can also be shown by using a simple Linear Regression Model (Fig. S18, S19). In Sec. Supplementary Note 8, we prove the robustness of our results with respect to the underlying dataset

(Fig. S20, S21) and the chosen community detection algorithm (Fig. S22, S23).) We provide further complex network analyses in Sec. Supplementary Note 9. Different network measures are analyzed globally (Fig. S24-S26) as well as community-specific (Fig. S27-S30). We provide a further discussion on the MJO similarity in Sec. Supplementary Note 10 (Fig. S31). More detailed information to the connection the El Niño Southern Oscillation (ENSO) is given in Sec. Supplementary Note 11 (Fig. S32, S33). We also show classical conditions for different ENSO types in boreal summer (Fig. S34). We also visualize the effect of the ENSO condition on the vertical velocities over the Maritime Continent for the 3 propagation modes (Fig. S35) To provide the reader with background to the current discussion in the literature, we outline the basic concepts of the moisture mode theory (Sec. Supplementary Note 12, Fig. S36, S37) and the vertical shear mechanism (Sec. Supplementary Note 13, Fig. S38). In the main text, we focused mainly on the BSISO-dominated communities. A detailed discussion on the North India-China region and its connection to current literature is provided in Sec. Supplementary Note 14 (Fig. S39, Fig. S40, Fig. S41).

Supplementary Note 1 Extreme rainfalls in the Indo-Asia Pacific domain

To construct the climate network of EREs in the Indo-Asia Pacific region, we only take into account those grid locations where the 95th percentile value of rainfall is greater than 10 mm/day or if we find more than 10 such 'event days' over the whole time period. Regions around the Arabian Peninsula and the Gobi and Taklamakan deserts were thus excluded from the analysis (compare Figure S1). We find that number of extreme events varies significantly between the tropics and the subtropics, with the tropics experiencing a far higher number of events (Figure S1). Our null model (sec. Methods) which depends on the number of events in each time series, helps to reduce the bias introduced in the event synchronization measure due to different event rates[1]. The upper bound on the dynamical delay is set to $\tau_{\max} = 10$ days for all grid location pairs.

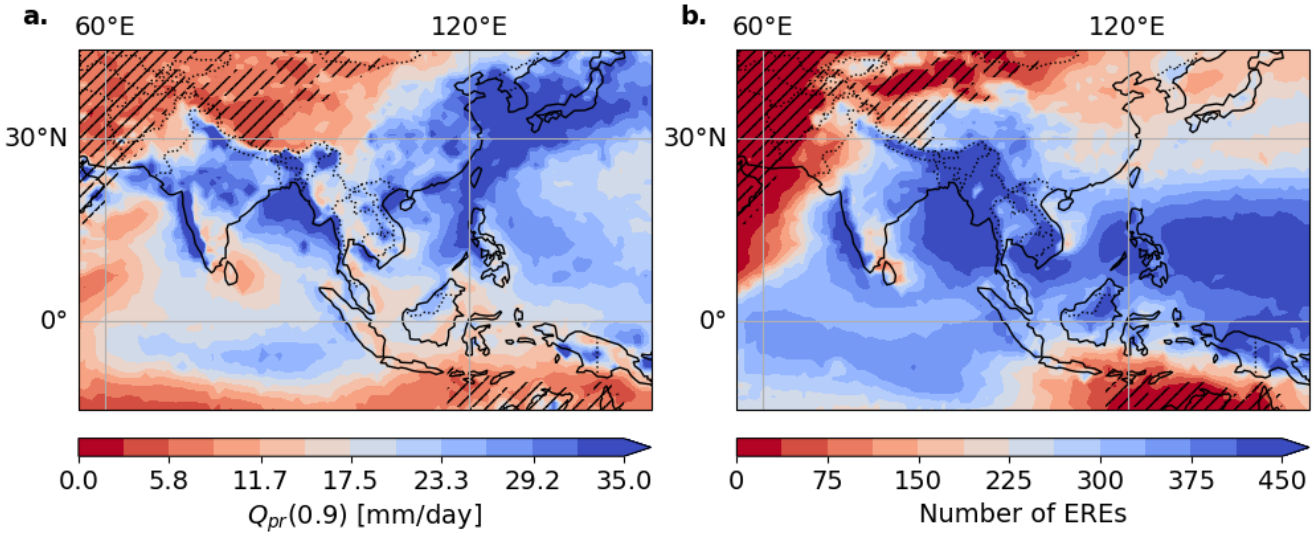

Figure S1. Quantiles and total numbers of extreme-rainfall events. a yields the number of counted EREs per spatial location. **b** shows the respective values of the quantile local function $Q_{0.9}$ for each location. Data is used from the MSWEP dataset[2] over the period from 1979–2021. An Extreme Rainfall Event (ERE) is defined as a day with a rainfall sum more than the 90th percentile of all wet days (i.e. days with rainfall more than 1 mm/day. Locations with less than 10 events in total are excluded from the analysis and marked as hatched areas.

Supplementary Note 2 Event Synchronization and network construction

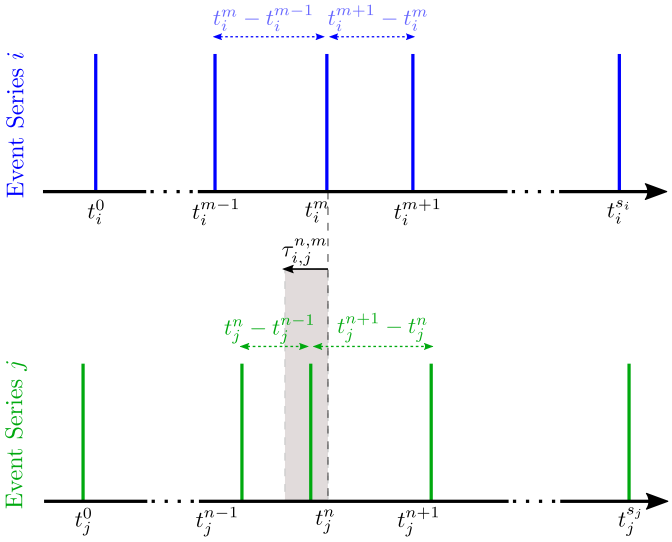

Figure S2. Scheme of time-delayed Event Synchronization. The point in time t_i^m is identified as synchronous to t_j^n since the time difference between t_i^m and t_j^n is within the allowed the range given by $\tau_{ij}^{m,n}$ according to the definition expressed in equation (see Methods). Not shown here is the maximum delay $\tau_{\max}$.

Fig. S2 describes the ES scheme. Note that if two or more events in one series occur at subsequent time steps the dynamical delay $\tau_{i,j}^{m,n}$ will result in a value of 1/2, leading to a case where it is likely that two sequentially occurring events are not recognized as synchronous. Therefore, in our analysis, blocks of consecutive events are counted as one event, placed on the point in time of the first event.

Supplementary Note 2.1 Event Synchronization based network construction

The construction of the network in our study is based on the event synchronization technique, which enables the estimation of statistically significant synchrony between events across different time series. This technique allows us to quantify the point-wise similarity of extreme event time series.

One key element is the adjacency matrix A, which holds significant importance in describing the network mathematically. The adjacency matrix is a square matrix of size $N \times N$, where N represents the number of spatial locations, equivalent to the number of time series under consideration. In this matrix, if $A_{i,j} = 1$, it indicates that events at location i are statistically significantly followed by events at location j. To aid in the interpretation of the adjacency matrix and provide a more intuitive understanding, we provide a schematic of the network construction in Fig. S3.

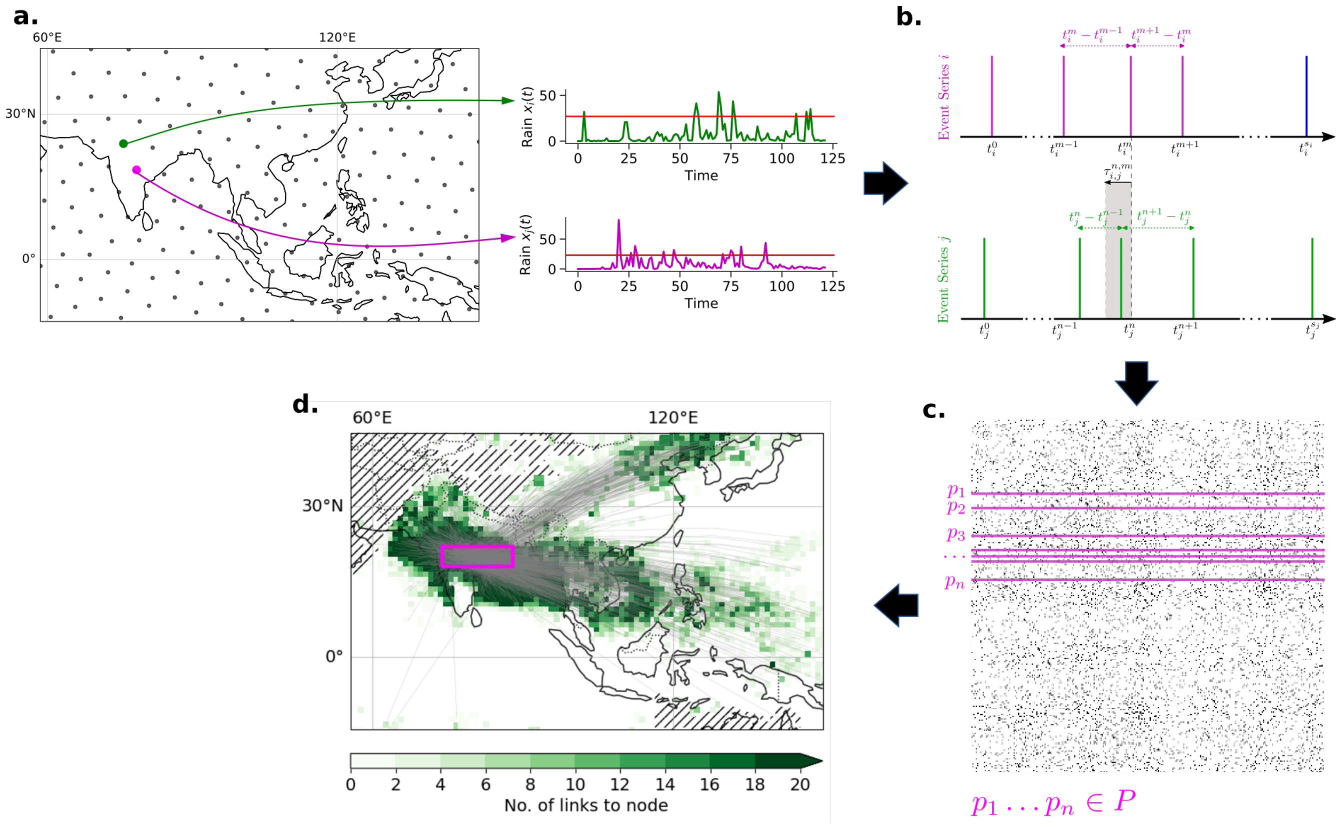

Figure S3. Scheme of network construction and interpretation using event synchronization. a Time series of precipitation data. These translate to event time series. **b** The point in time t_i^m is identified as synchronous to t_j^n since the time difference between t_i^m and t_j^n is within the allowed range given by $\tau_{ij}^{m,n}$ according to the definition (see Methods). **c** The network is constructed by point-to-point comparison of all time series to each other and mathematically expressed by the $N \times N$ adjacency matrix A, where N denotes the number of locations. **d** The highlighted rows of the adjacency matrix A translate to a set of points P (pink rectangle) that represent spatial locations that are statistically significantly synchronous to further locations (the black dots in the respective row) to network links.

Supplementary Note 3 BSISO drives the organization of synchronous EREs

We compute the membership likelihood for the 6 identified monsoon regions in Fig. 2 a. These are computed as it is described in Sec. . The membership likelihoods for the different climate network communities are visualized in Fig. S4. These plots show low spatial variability of the communities and therefore underline the robustness of the community detection algorithm.

The BSISO is directly reflected in the shape of the communities. The BSISO is known to emerge around the center of the Equatorial Indian Ocean characterized by intense rainfalls[3,4] consistent with our observation of an increased likelihood of synchronous EREs in the EIO community for BSISO phases 1 and 2 (Fig. 3 a)[5]. The BSISO-driven rainfalls are known to intensify significantly over the Bay of Bengal[6] consistent with our observations (Fig. 3 c). The increased probability of experiencing extreme precipitation during active BSISO by a factor of two to three in the MC community region is consistent with observations in other studies[7]. The strongest enhancement in SA occurs during BSISO phases 4 to 6 (Fig. 3 d) establishing a monsoon trough developing here over the Bay of Bengal and leading to winds blowing southwesterly across the Indian Ocean carrying a lot of moisture. Many studies suggest that the establishment of a such convergence zone along the monsoon trough is enhanced during active BSISO expressed by strong low-pressure systems[6,8–12]. In WP, EREs occur during BSISO phases 6 and 7 (Fig. 3). The intraseasonal variation of the convection anomalies over the tropical eastern Indian Ocean until the western Pacific Oceans, so-called Pacific Japan (JP)-mode, has been attributed to the summertime MJO[13].

For each of these regions we derive a community-specific synchronous rainfall index (SRI) (see Methods, Fig. 1). This index can also be used to determine the most synchronous days per community (Fig. S5). Using the synchronous rainfall index (Fig. S5), it is also possible to derive the power spectrum of each community-specific synchronous rainfall index. We compare it to an Autoregressive Model with lag 1 autocorrelation (AR(1)). We find that the characteristic 30-60 day period of the BSISO is also dominant for all communities except the North-India China community indicating that this community is not dominated by the BSISO (Fig. S7).

We can also show the BSISO propagation using Hovmöller diagrams for the most synchronous days in the EIO community. In Fig. S8, we observe an eastward propagation via the Maritime Continent and a northward propagation. However, the propagation pattern gets blurry around 5-10 days after the initiation suggesting a variability in the propagation pathways.

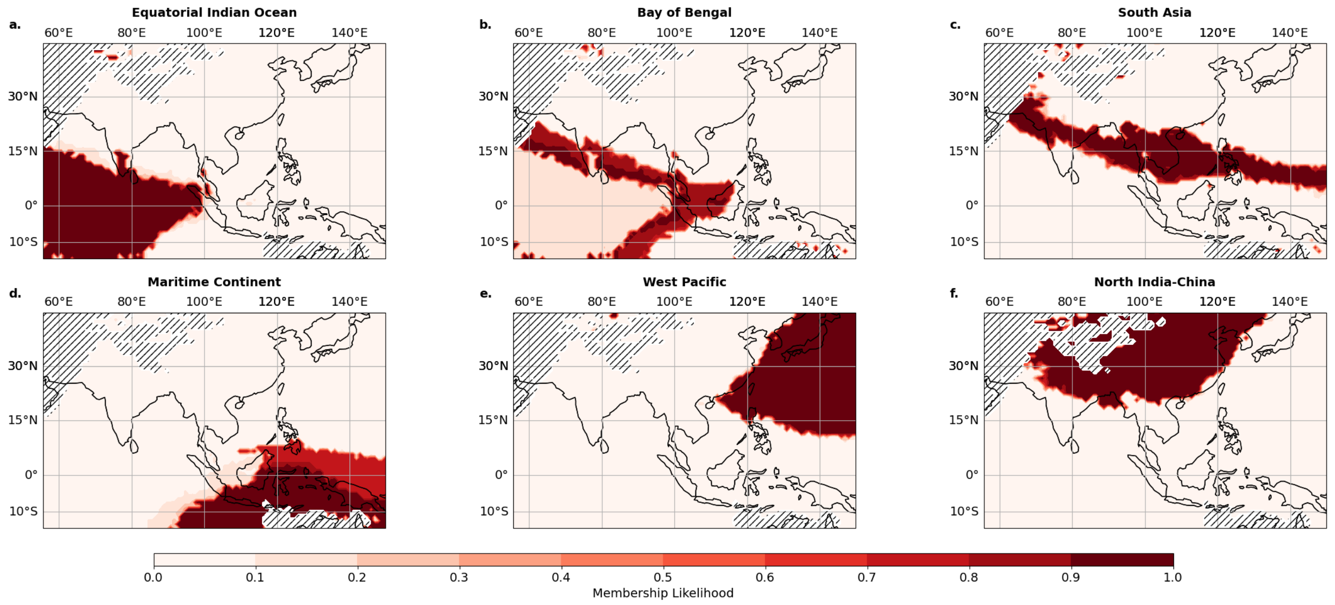

Figure S4. Membership Likelihoods for different communities. Using the heuristic outlined in sec.   we find 6 stable communities. The colorbar shows the membership likelihood of a respective community. 100 independent runs of the community detection algorithm have been used for this analysis.

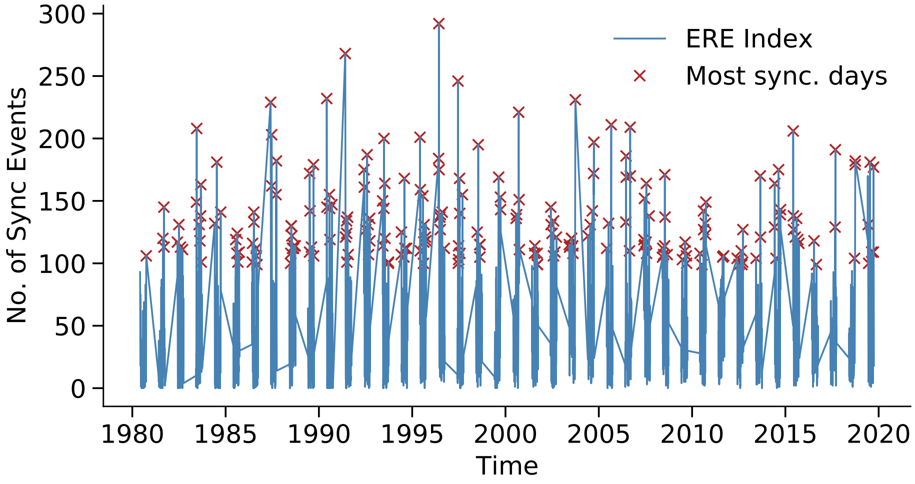

Figure S5. Definition of synchronous extreme rainfall event index. The synchronous extreme rainfall event index (ERE index) (see Methods) is shown here as exemplary for the community in the Equatorial Indian Ocean (Fig. 2). We derive the most synchronous days by taking the peaks of all values above the 90th percentile denoted by red crosses.

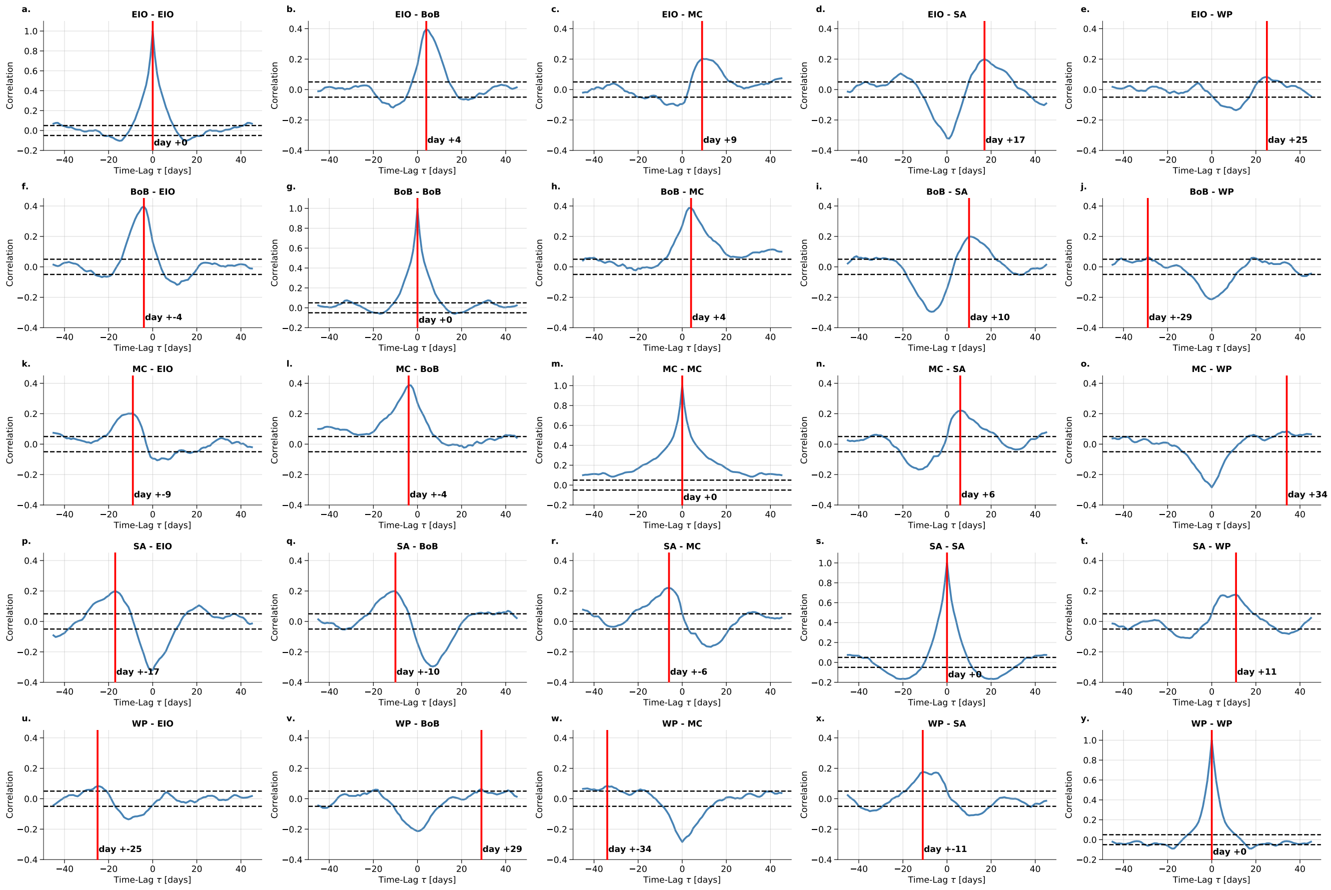

Figure S6. **Lead-Lag analysis for different communities.** Using the synchronous rainfall index (see Methods) we derive lead-lag correlation analysis based on Spearman's rank order correlation. The dashed line denotes the 0.95 confidence interval. Vertical solid red lines denoting the day of maximum correlation.

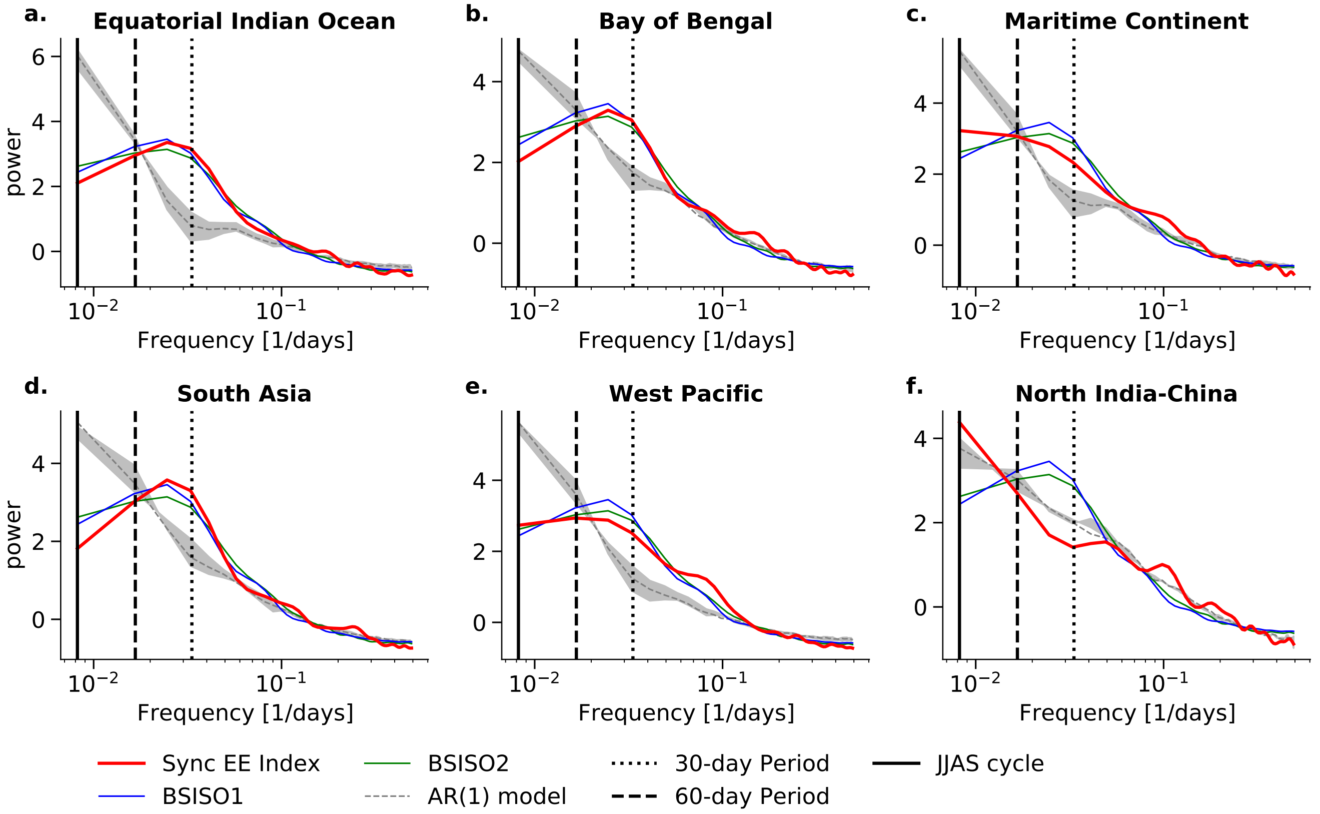

Figure S7. Power spectrum analysis of community-specific synchronous ERE index. Using the synchronous rainfall index (Fig. S5), we derive a power spectrum analysis for each community of its community-specific index (red line). It is compared to samples of an Auto-Regressive model with lag 1 autocorrelation (AR(1)). The dashed line denotes the mean and the grey band the 0.95 confidence interval of the AR(1) model. Vertical lines denoting the JJAS frequency and the dominant 30-60 day period of the BSISO. For comparison also the power spectrum of the BSISO1 (blue line) and BSISO2 (green line) index[14] are shown.

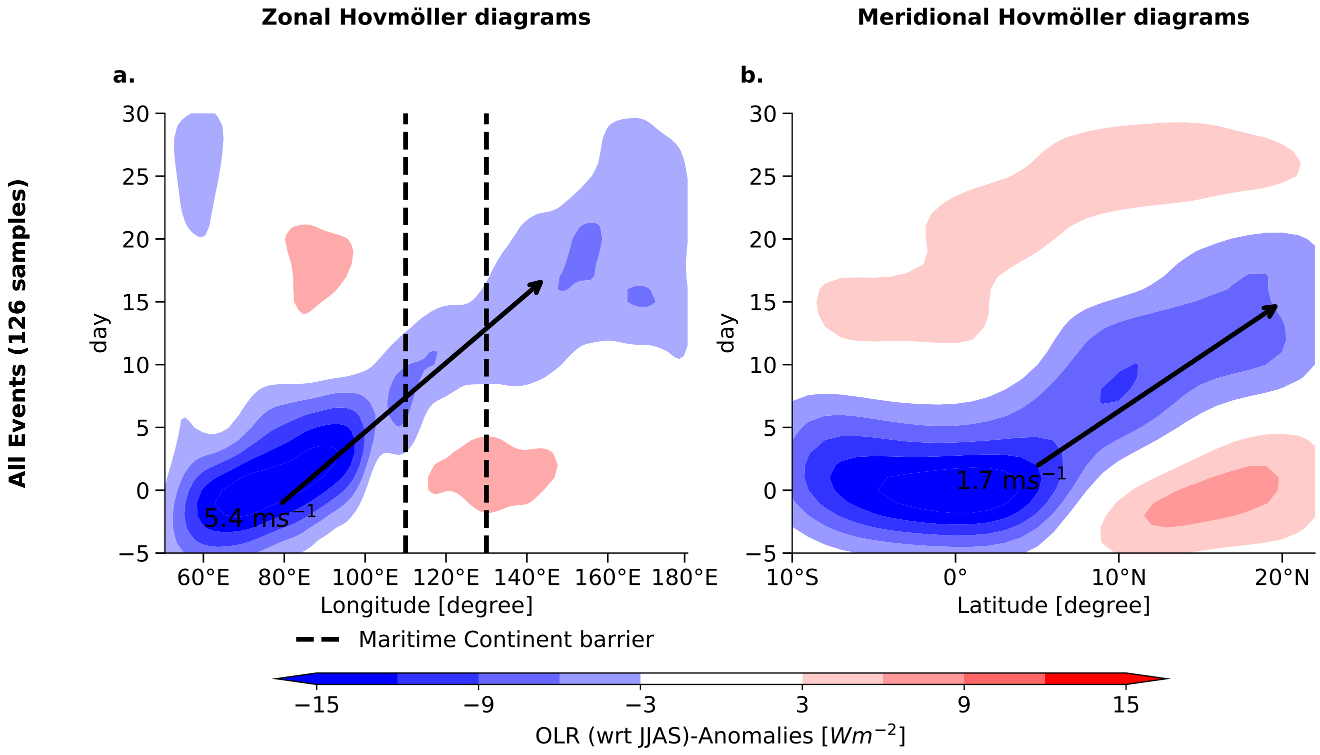

Figure S8. Hovmöller diagrams after initiation. The first column (**a**) shows the composited Hovmöller diagrams in the zonal direction, and the second column (**b**) in the meridional direction. All anomalies are computed with respect to the JJAS seasonality. Day 0 describes the days of maximum synchronization within the EIO community (Fig. 2 a). The dashed lines mark the area of the Maritime Continent barrier roughly estimated to be from 110° E-130° E and the arrows denote the estimated speed of the convective system.

Supplementary Note 4 Intra-annual distribution of occurrence of synchronous EREs

We count the occurrence of EREs for a day of maximum synchronization for the 6 identified monsoon regions in Fig. 2 a. Using the synchronous rainfall index (Fig. S5) per community it is possible to determine the average number of EREs per month (week) for a specific community, shown in Fig. S9 for the monthly distribution and Fig. S10 for the weekly distribution

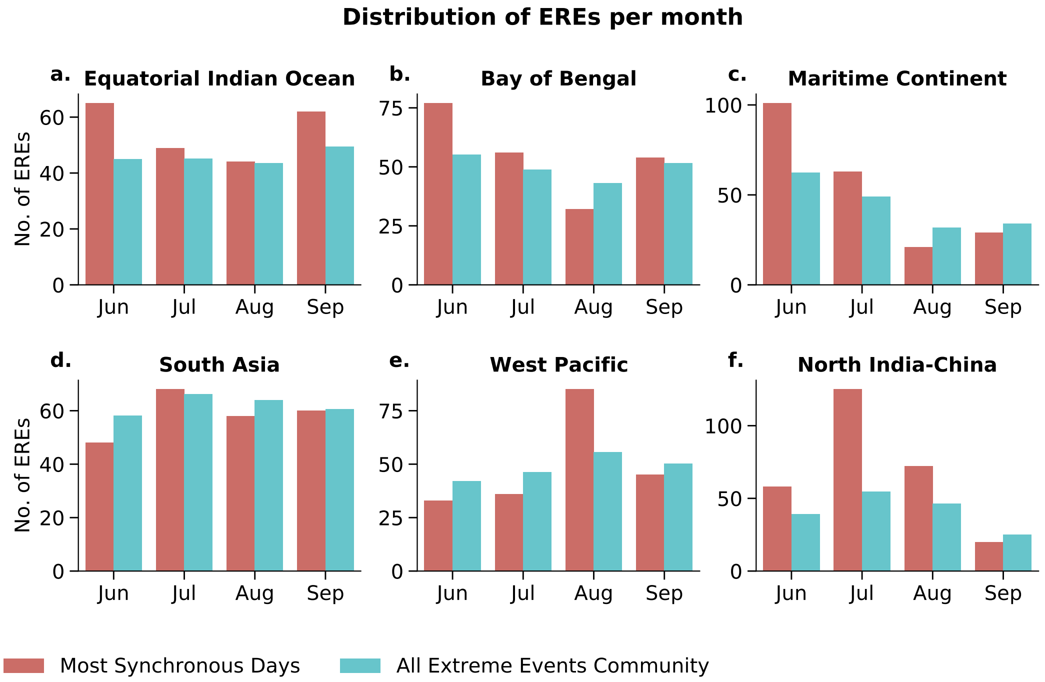

Figure S9. Histogram of the occurrence of EREs per month for different communities. Using the synchronous rainfall index (see Methods) we count the average number of EREs per day of maximum synchronization (red) and for all EREs that are in the community (green) normalized by the number of days.

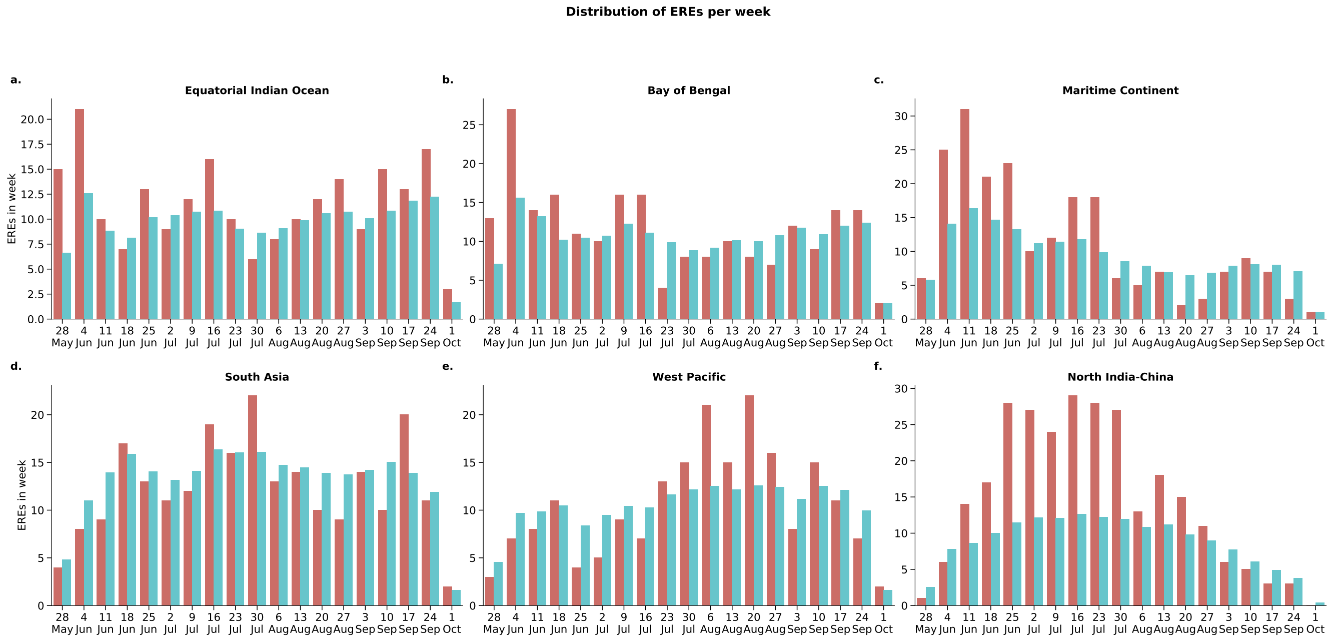

Figure S10. **Histogram of the occurrence of EREs per week for different communities.** Using the synchronous rainfall index (see Methods) we count the average number of EREs per day of maximum synchronization (red) and for all EREs that are in the community (green).

Supplementary Note 5 Propagation pattern of EREs associated with BSISO

Supplementary Note 5.1 Single events

In this subsection, for illustrative purposes, we investigate the propagation of individual Boreal Summer Intraseasonal Oscillation (BSISO) events across the Indian Ocean, the Maritime Continent, South Asia, and India, extending towards the Western Pacific specifically for the year 1990 to relate the propagation of EREs to the precipitation anomalies. By analyzing this selected year, we aim to provide a comprehensive understanding of the spatiotemporal dynamics and trajectories of BSISO-related events, shedding light on their propagation patterns.

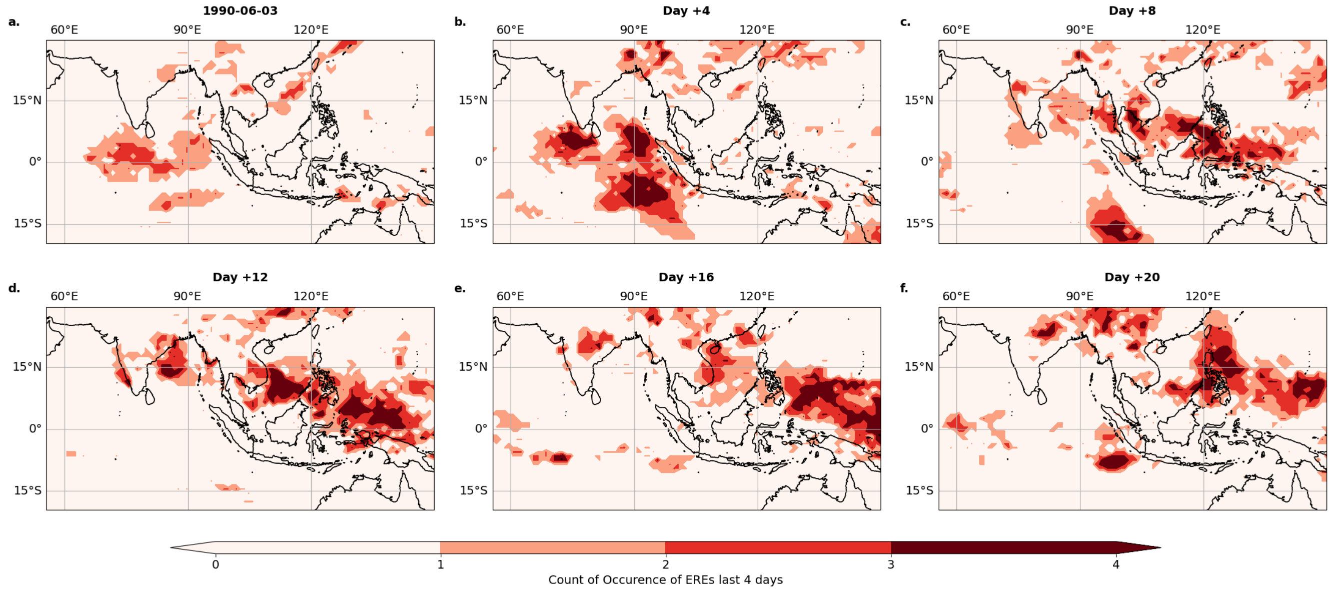

Figure S11. Propagation of Boreal Summer Intraseasonal Oscillation (BSISO) Events in terms of EREs for 1990. EREs are estimated following the methodology described in SI sec. Supplementary Note 1. The spatial evolution of BSISO-related ERE events for the year 1990 is shown. Each plot shows the accumulated sum of the occurrence of EREs in the last 4 days.

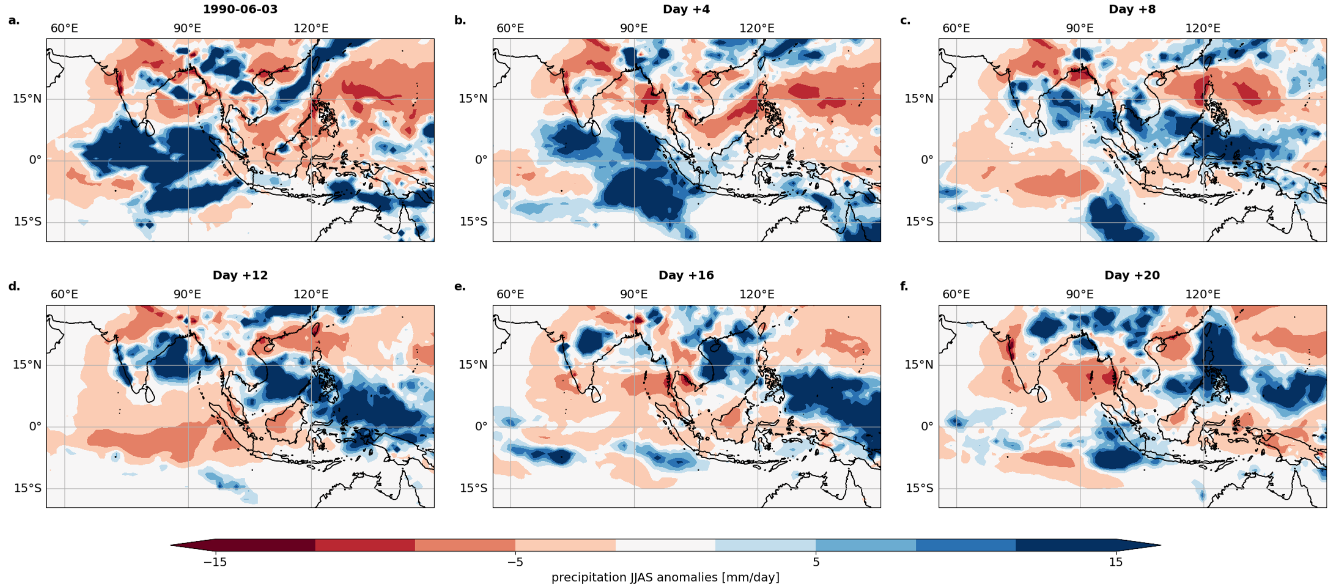

Figure S12. Propagation of Boreal Summer Intraseasonal Oscillation (BSISO) Events in terms of precipitation anomalies for 1990. The spatial evolution of a BSISO-related event of the year 1990 is shown here in terms of precipitation anomalies with respect to the JJAS climatology. The points in time are the same as in Fig. S11

Supplementary Note 5.2 BSISO propagation modes

The propagation of the BSISO is subdivided into 3 different modes. The propagation of the convective system is shown in a condensed way in Fig. 9. Here, we show spatial composites of all three propagation modes in their spatial progression until 24 days in advance. The Canonical propagation is shown in Fig. S13, the Eastward Blocked mode in Fig. S14, and the Stationary case in Fig. S15.

These propagation pathways also translate to propagation of EREs along the previously identified communities (Fig. 2 a). The Canonical propagation is shown in Fig. S16.

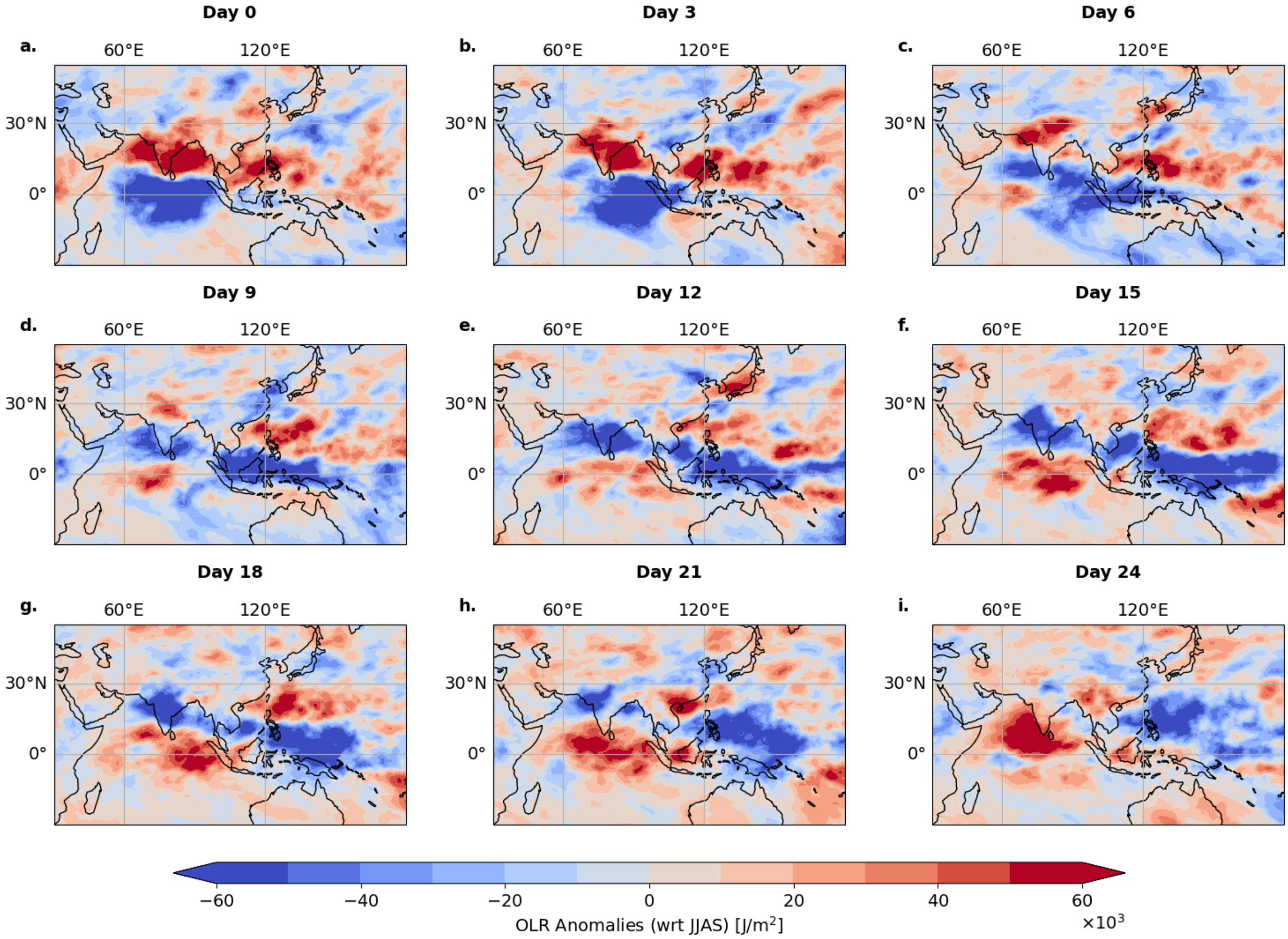

Figure S13. **Propagation of anomaly precipitation for Canonical BSISO propagation mode.** The rainfall anomalies are computed according to the JJAS climatology. Day 0 denotes the days of maximum synchronization in the EIO community (Fig. 2 a) classified as "Canonical" BSISO propagation mode (Fig. 4 2nd row). The following plots **b-i** are the composites of the respective days after day 0 in steps of 3 days.

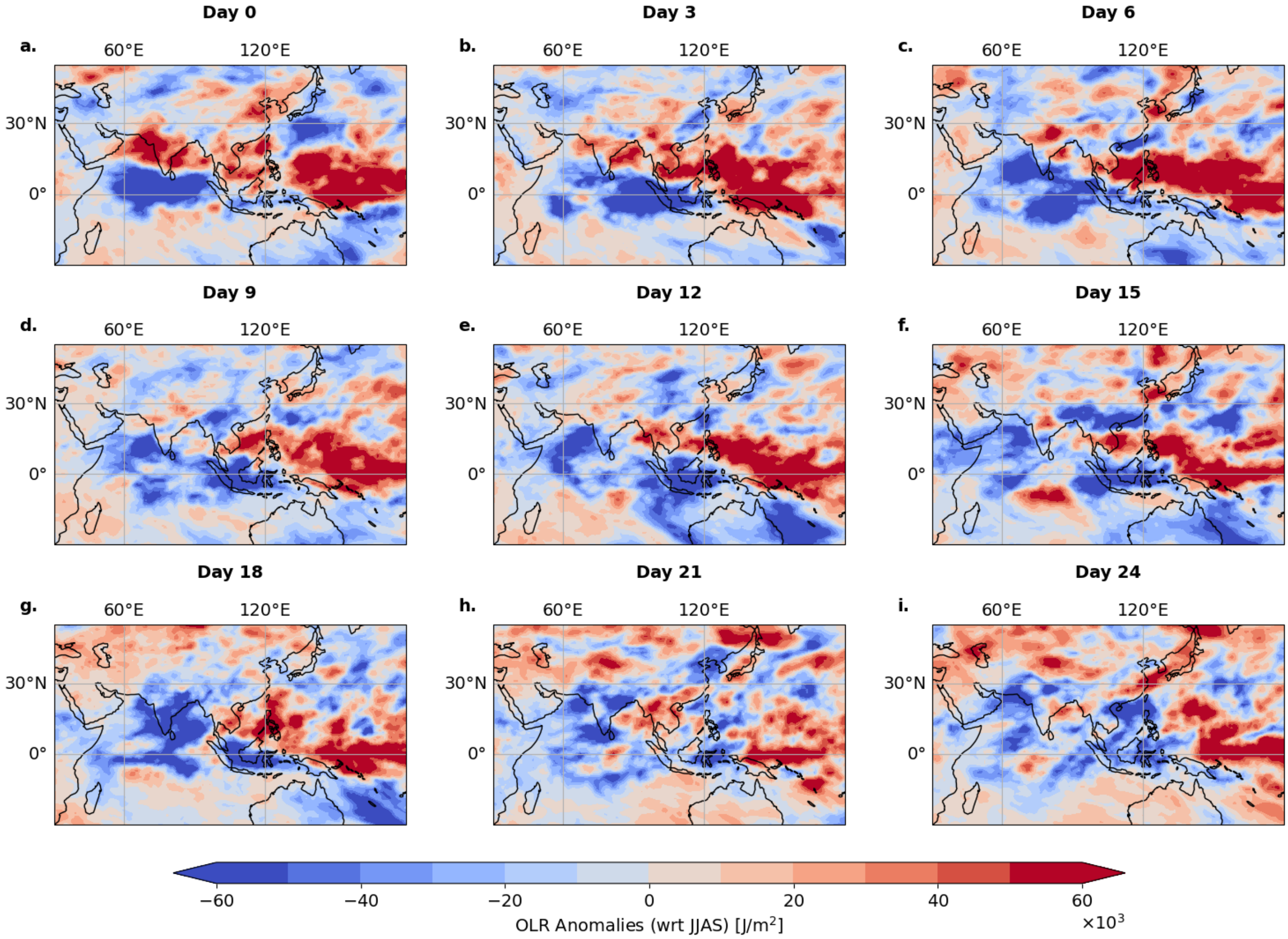

Figure S14. Propagation of anomaly precipitation for Eastward Blocked BSISO propagation mode. The rainfall anomalies are computed according to the JJAS climatology. Day 0 denotes the days of maximum synchronization in the EIO community (Fig. 2 a) classified as "Eastward Blocked" BSISO propagation mode (Fig. 4 2nd row). The following plots **b**-**i** are the composites of the respective days after day 0 in steps of 3 days.

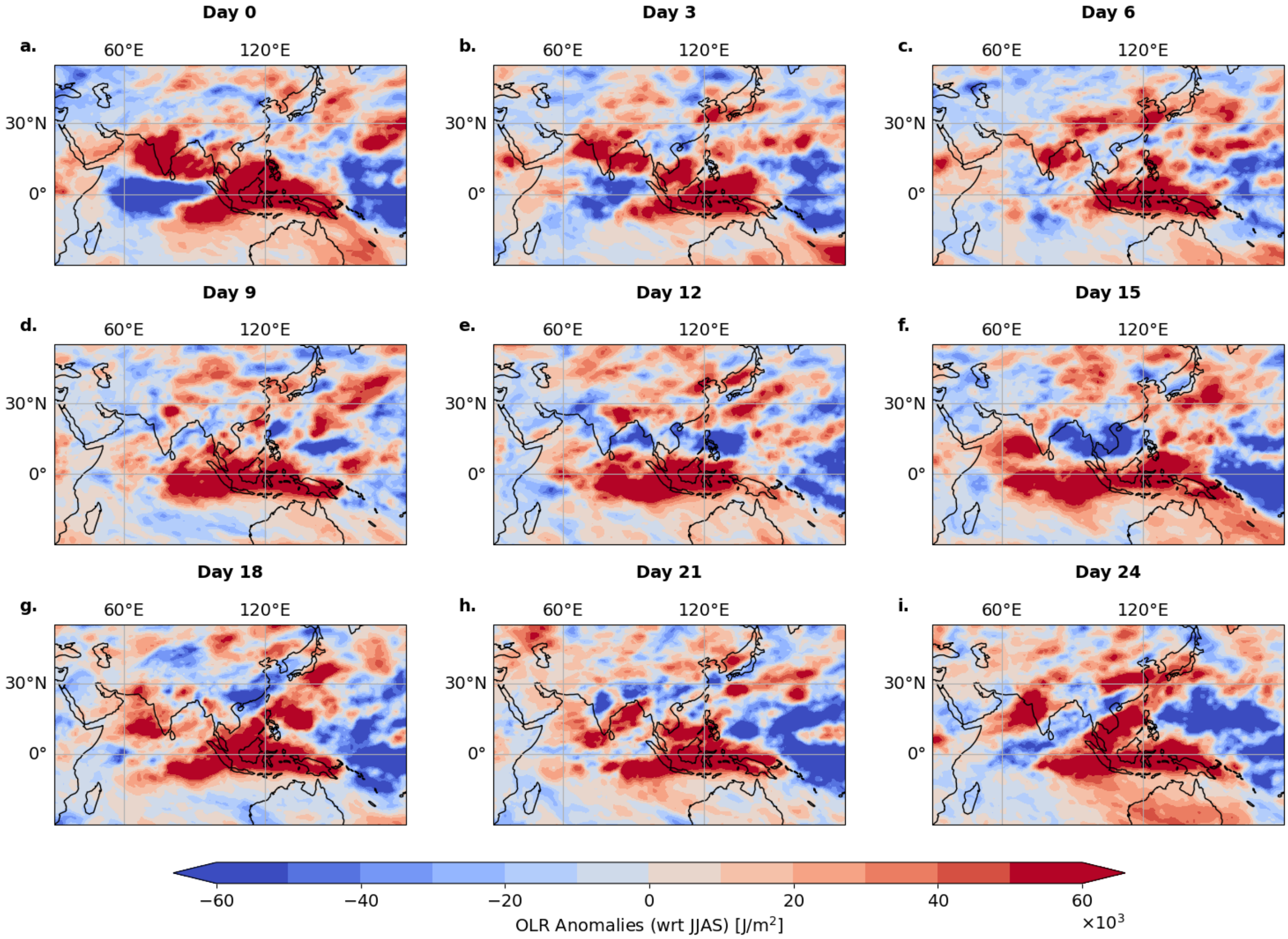

Figure S15. Propagation of anomaly precipitation for Stationary BSISO propagation mode. The rainfall anomalies are computed according to the JJAS climatology. Day 0 denotes the days of maximum synchronization in the EIO community (Fig. 2 a) classified as "Quasi-stationary" BSISO propagation mode (Fig. 4 3rd row). Rainfall anomalies occur solely in the region of the EIO community.

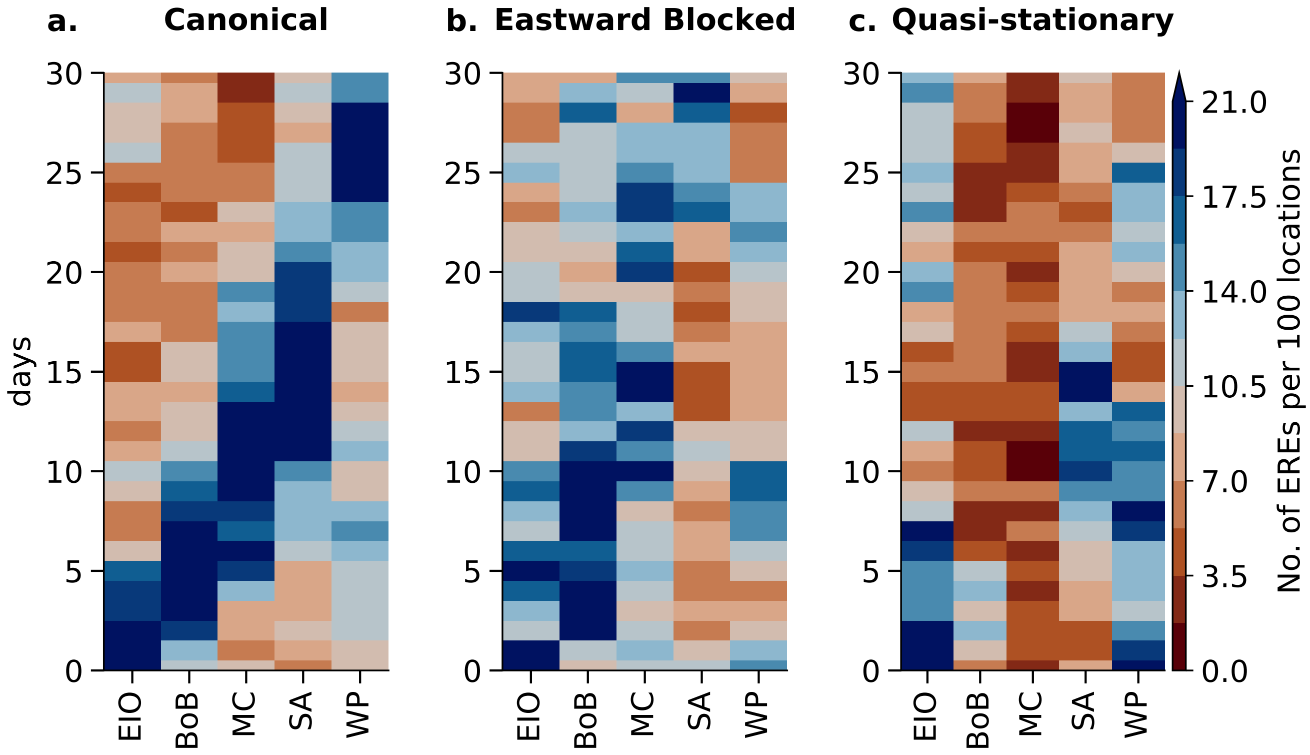

Figure S16. **Propagation of EREs along communities.** The rainfall extremes are computed according to section Supplementary Note 1. Day 0 denotes the days of maximum synchronization in the EIO community (Fig. 2 a). **a** shows BSISO propagation events classified as "Canonical", **b** events classified as "Eastward blocked" and **c** events classified as "Quasi-stationary".

Supplementary Note 6 Influence of the choice of BSISO indices

There are multiple indices that describe the characteristics of the BSISO propagation. While the index defined by[14] which is used in this study is better suited for tracking BSISO convection[15], the RMM index[16] and the BSISO index by[17] is better describing the circulation associated with BSISO when convection is reduced[15]. Here, we show that our qualitative results from Fig. 3 remain unaffected by the choice of the specific BSISO index. Fig. S17 shows the distribution over phases using the index described by[17]

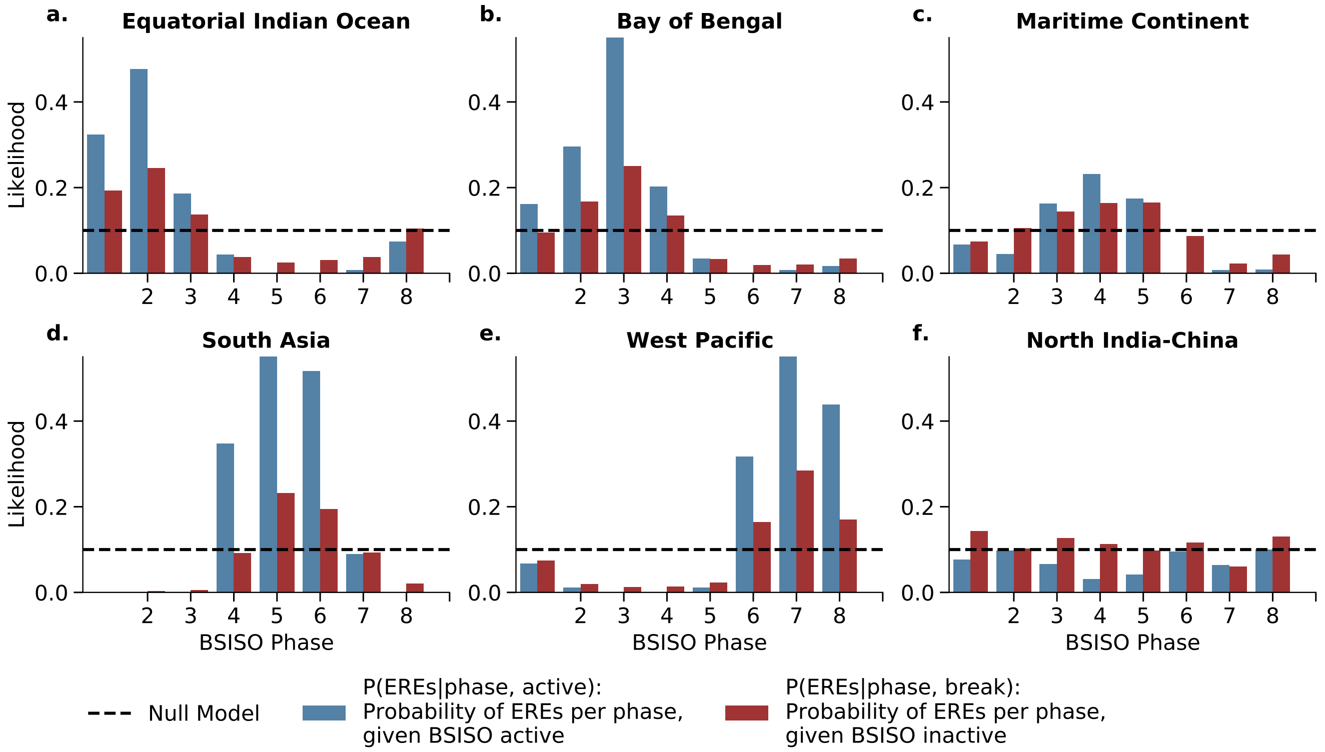

Figure S17. Likelihood of synchronous events for active/inactive BSISO phases. The likelihood of the occurrence of synchronous events ($s = 1$) is analyzed for active (blue) and inactive (red) BSISO phases (as defined by[17]) in the regions of Fig. 2 a: **a** equatorial Indian Ocean, **b** Bay of Bengal, **c** Maritime Continent, **d** South Asia, **e** Western Pacific, and **f** North India-China. The dashed line illustrates the likelihood of synchronous events estimated from a null model of randomly distributed synchronous events (i.e. by construction 10 %).

Supplementary Note 7 Correlation and linear model for BSISO index

The community-specific time series are statistically significantly correlated to the two components of the BSISO index using both the definition by[14] (Fig. S18) or the by[17] (Fig. S19). The time series of the communities EIO, BoB, MC, SA and WP are statistically significantly correlated with either or both the BSISO1 and BSISO2 index. The NIC ERE index is not significantly correlated.

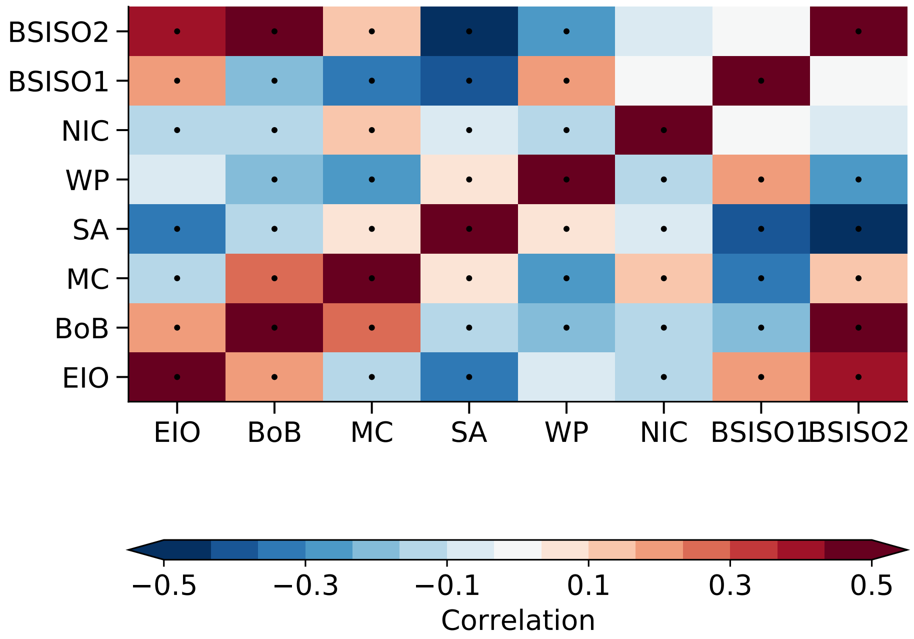

Figure S18. Correlation matrix of community-specific ERE index and BSISO index. The correlation of the community-specific ERE index and the two components of the BSISO index (as defined by Kikuchi et al.[14]) for the regions of Fig. 2 a: Equatorial Indian Ocean (EIO), Bay of Bengal (BoB), Maritime Continent (MC), South Asia (SA), Western Pacific (WP), and North India-China (NIC). Dots indicate significant correlations at the 99% confidence level using Student's t-test.

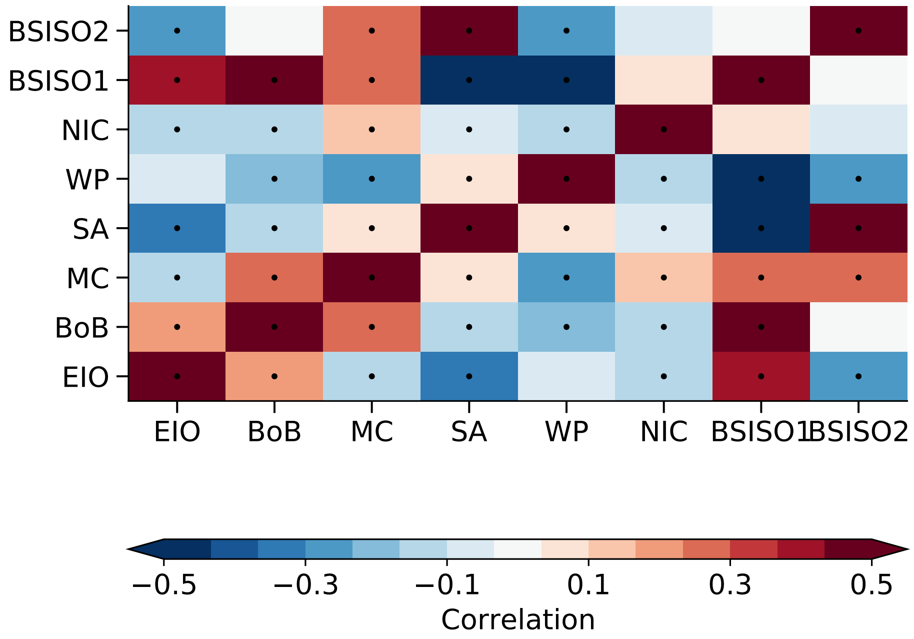

Figure S19. Correlation matrix of community-specific ERE index and BSISO index. The correlation of the community-specific ERE index and the two components of the BSISO index (as defined by Lee et al.[17]) for the regions of Fig. 2 a: Equatorial Indian Ocean (EIO), Bay of Bengal (BoB), Maritime Continent (MC), South Asia (SA), Western Pacific (WP), and North India-China (NIC). Dots indicate significant correlations at the 99% confidence level using Student's t-test.

The consistency between the community-specific ERE indices and the BSISO index can also be shown by a linear model. The model is estimated in the following way: A target time series $Y(t)$ is modelled by N input time series $X_i(t)$, where $i = 1, \ldots, N$. We use a simple multi-dimensional linear regression incorporating time lags τ_i, expressed as:

$$Y(t) = \sum_{i=0}^{N} a_i X_i(t - \tau_i) + b + \varepsilon(t) \ , \tag{1}$$

where $a_0 \ldots a_N, b$ are the fitting parameters and $\varepsilon(t)$ is the error term. The fitting parameters are estimated using the least square fit. The goodness of the fit (also denoted as "explained variance") is expressed by the square of the correlation between the observed Y values and the predicted $\hat{Y}$ values as $r^2 = \sum(\hat{Y}(t) - \overline{Y}(t)/Y(t) - \bar{Y}(t))$, where $\overline{Y}$ denotes the time average.

In order to quantify the amount of ERE variability that is explained by BSISO, we use the simple linear regression model between specific synchronous ERE indices (see Material&Methods Sec.) and the PC_1 and PC_2 indices of the BSISO index Kikuchi et al.[14]. We obtain the following explained variances: EIO $r = 67$ %, BoB $r = 77$ %, SA $r = 78$ %, MC $r = 54$ %, WP $r = 70$ % and NIC $r = 8$ %. Conversely, BSISO variability is explainable from the community synchronization indices with explained variances of $r = 83$ % ($r = 68$ %) for PC_1 (PC_2) indices. These results, obtained by using a simple linear regression (and applying a low-pass filter with 3 days cutoff on the time series to neglect small daily variations), affirm the close relationship of ERE synchronization to the BSISO.

Supplementary Note 8 Robustness tests

In this section, we show that our results are robust to variations of the choices presented in the main text on either the underlying dataset (sec. Supplementary Note 8.1) or the employed community detection algorithm (sec. Supplementary Note 8.2)

Supplementary Note 8.1 Sensitivity to dataset

To assess the robustness of our findings, we conducted a comparative analysis using the Tropical Rainfall Measuring Mission (TRMM) dataset[18], which provides global coverage with a spatial resolution of 0.25×0.25. The TRMM dataset was processed for the same spatial region and interpolated to match the resolution of the Fekete grid employed in the MSWEP dataset (see Section). By comparing the results presented in Figure 2 a obtained with the MSWEP dataset with the communities identified using the TRMM dataset (Fig. S20), we observe qualitatively similar spatial patterns. However, the membership likelihoods exhibit slightly larger variability in the TRMM dataset (Figure S21) compared to the MSWEP dataset (Figure S4). This discrepancy can be attributed to the shorter time length of the TRMM dataset, which reduces the statistical robustness of the climate network links. Notably, the Bay of Bengal (BoB) community exhibits the largest difference between the MSWEP and TRMM datasets.

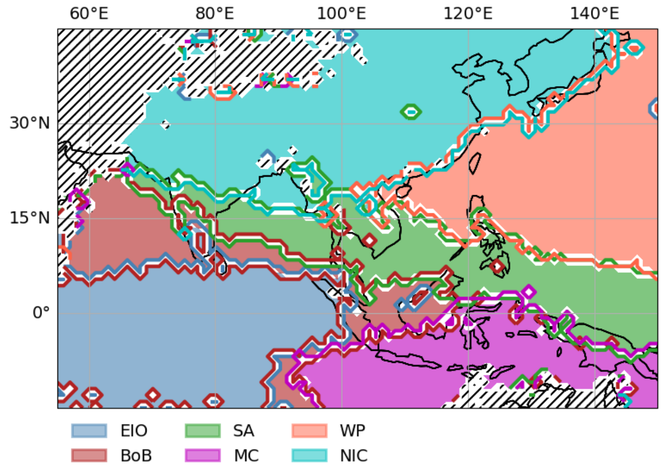

Figure S20. Membership Likelihoods for different communities using the TRMM dataset. Using the heuristic outlined in sec. we find 6 stable communities of a climate network that is constructed by using rainfall data from the TRMM dataset. These are labeled according to their spatial mean position, i.e. equatorial Indian Ocean (EIO), Bay of Bengal (BoB), Maritime Continent (MC), South Asia (SA), Western Pacific (WP), and North India-China (NIC). The color always shows the community for a respective location with its highest membership likelihood that comprises 100 independent runs of the community detection algorithm that has been used for this analysis. Hatched areas indicate regions with too little precipitation, which are excluded from the analysis and white regions indicate locations that cannot be assigned to a single community.

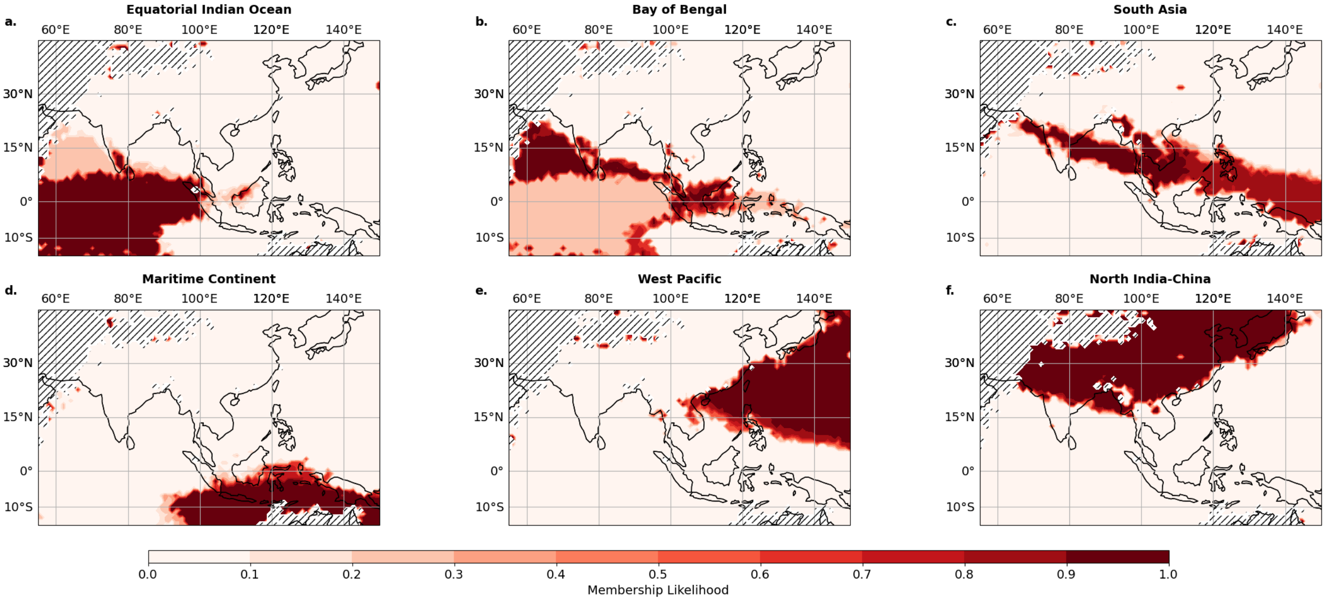

Figure S21. Membership Likelihoods for different communities using the TRMM dataset. Using the heuristic outlined in sec. we find 6 stable communities. The colorbar shows the membership likelihood of a respective community. 100 independent runs of the community detection algorithm have been used for this analysis.

Supplementary Note 8.2 Sensitivity to the choice of community detection algorithm

Here we employ the same climate network as used in the main text to a further community detection algorithm that uses the Parallel Louvain method[19] in the implementation of the NetworkIt package[20]. The observed communities exhibit a qualitative structure that is consistent with the results obtained by the SBM approach (Fig. 2 a). Nevertheless, we opted for the implementation of the Stochastic Block Model (SBM) due to its ability to yield more stable results in terms of the spatial configurations of the identified communities.

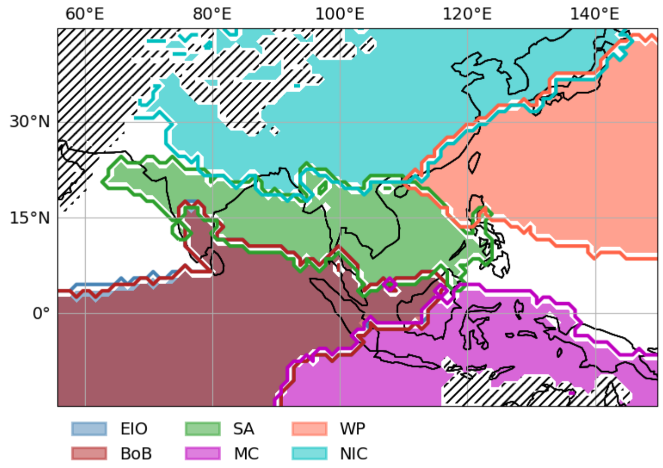

Figure S22. Different communities using PLM method implementation. Using the heuristic outlined in sec.   we find 6 stable communities of a climate network that is constructed by using rainfall data from the TRMM dataset. These are labeled according to their spatial mean position, i.e. equatorial Indian Ocean (EIO), Bay of Bengal (BoB), Maritime Continent (MC), South Asia (SA), Western Pacific (WP), and North India-China (NIC). The color always shows the community for a respective location with its highest membership likelihood that comprises 100 independent runs of the community detection algorithm that has been used for this analysis. Hatched areas indicate regions with too little precipitation, which are excluded from the analysis.

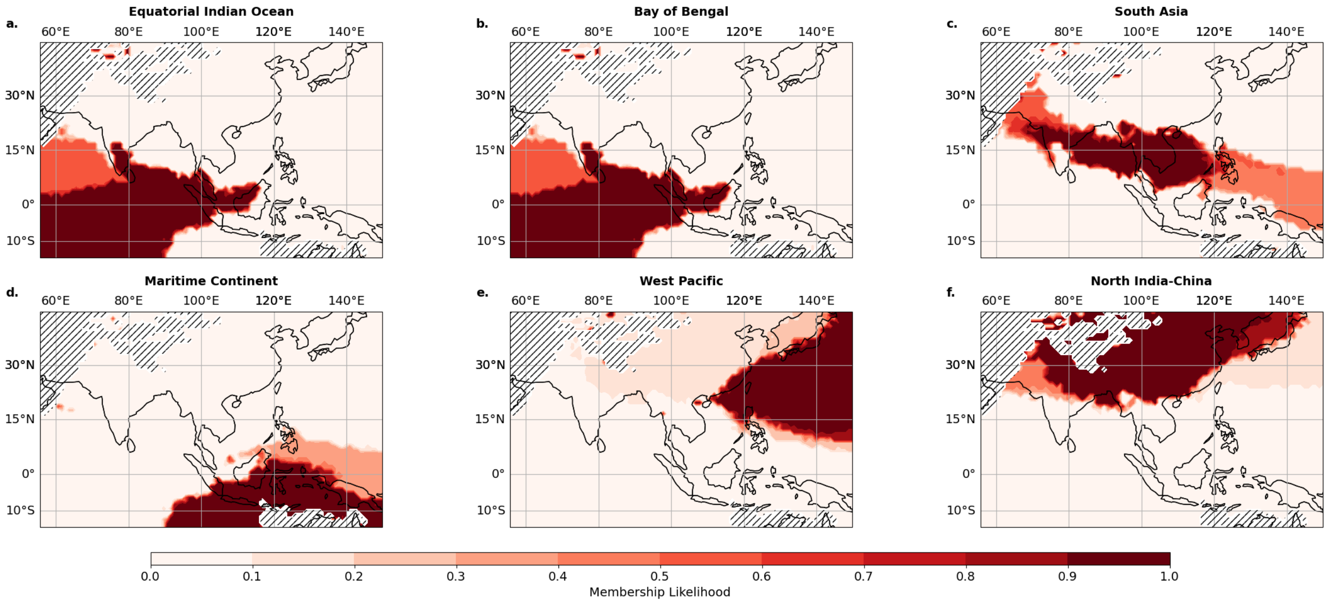

Figure S23. Membership Likelihoods for different communities using the MSWEP dataset with PLM community detection algorithm. Using the heuristic outlined in sec.   we find 6 stable communities. The colorbar shows the membership likelihood of a respective community. 100 independent runs of the community detection algorithm have been used for this analysis.

Supplementary Note 9 Network Analysis

The dynamics of different mesoscale systems and the boreal summer atmospheric circulation is reflected by the network structure, and recent studies have shown that different network metrics help to uncover these systems which has been applied in various contexts for analyzing monsoon-related rainfall dynamics (eg.[21–25]). The event synchronization algorithm applied might be very context-specific and differs from study to study. For example, both the spatial range and the time range differ when analyzing tropical cyclones[26] compared with long range teleconnections[21,25]. Similarly, the term "community" is understood differently, for example, sometimes it is used to describe certain pronounced patterns in network measures[27]. Still, different network measures can help to better understand the network communities.

We have applied an analysis for node degree (Fig. S24), clustering coefficient (Fig. S25), betweenness centrality (Fig. S26), and network curvature. All measures are boundary corrected[28].

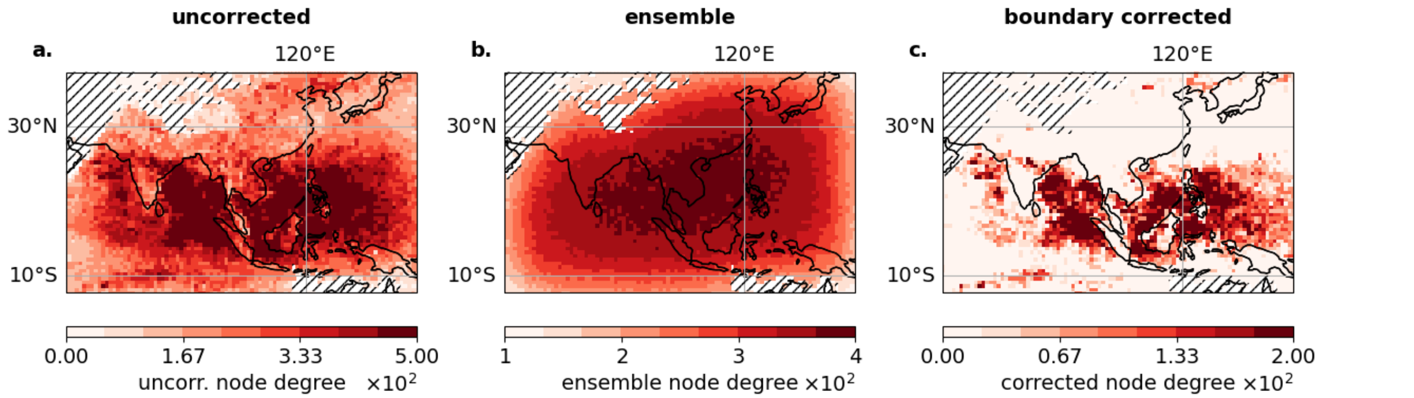

Figure S24. Node degree analysis. We investigate the node degree for the same network on which the community detection algorithm was applied (Fig. 2 a). We also visualize the effect of the boundary correction[28].

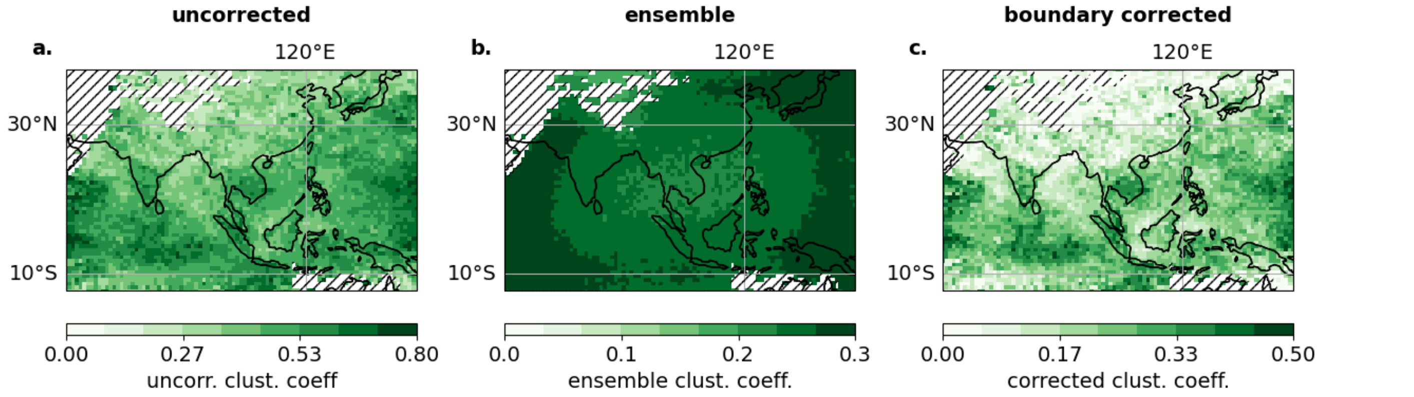

Figure S25. Clustering coefficient analysis. We investigate the clustering coefficient for the same network on which the community detection algorithm was applied (Fig. 2 a). We also visualize the effect of the boundary correction[28].

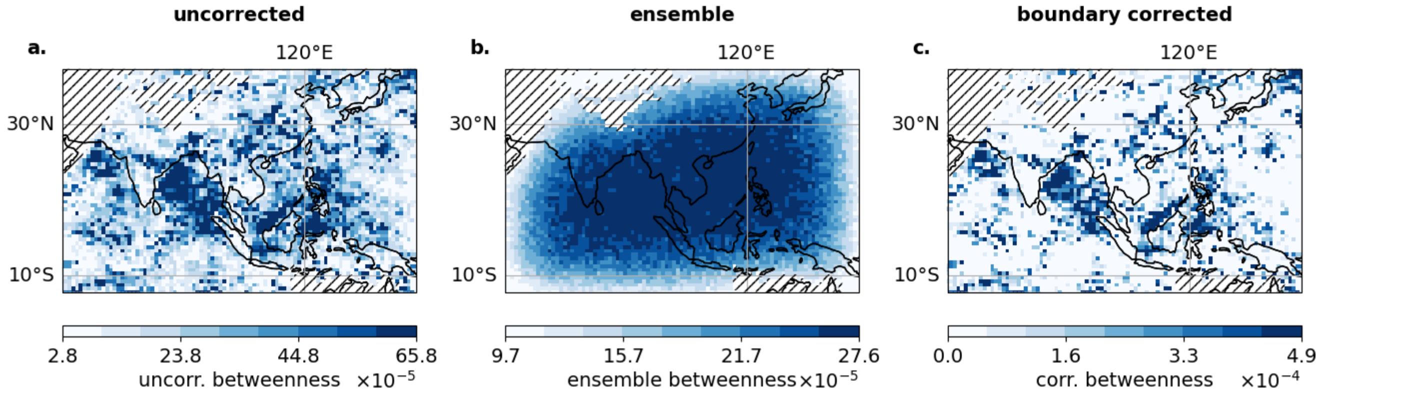

Figure S26. **Betweenness centrality analysis.** We investigate the betweenness centrality for the same network on which the community detection algorithm was applied (Fig. 2 a). We also visualize the effect of the boundary correction, using the method described in[28].

We further analyzed the community-specific network measures. Neither node degree (Fig. S27), nor betweenness centrality (Fig. S28) nor curvature (Fig. S29) provide substantial differences between the communities (Fig. 2 a). However, the local clustering coefficient (Fig. S30) between the NIC community and the other 5 communities shows a shifted distribution indicating that the density of the NIC is different to the other 5 communities. This might be related to the fact, that the community is mainly over land whereas the other communities cover substantial amounts over the ocean.

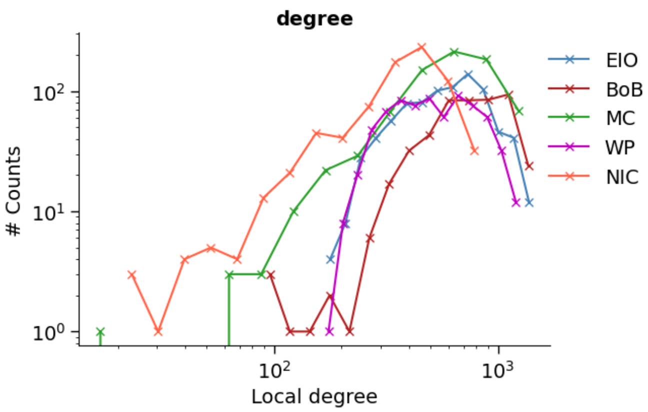

Figure S27. **histogram for node degree.** We investigate the node degree for the nodes of the network that are in the specific communities shown in Fig. 2 a.

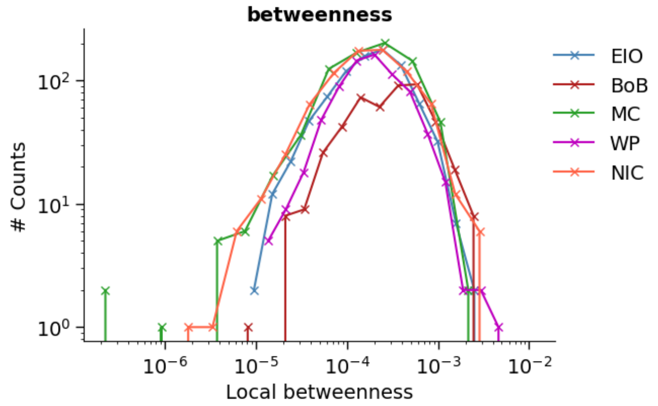

Figure S28. histogram for betweenness centrality We investigate the betweenness centrality for the nodes of the network that are in the specific communities shown in Fig. 2 a.

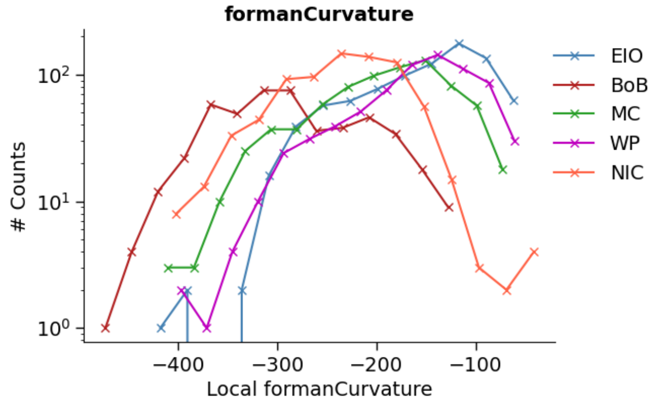

Figure S29. histogram for Forman Curvature We investigate the Forman Curvature for the nodes of the network that are in the specific communities shown in Fig. 2 a.

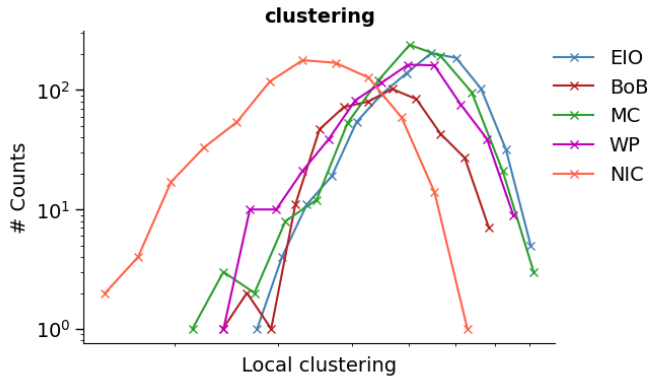

Figure S30. histogram for clusering coefficient. We investigate the local clustering coefficient for the nodes of the network that are in the specific communities shown in Fig. 2 a.

Supplementary Note 10 Comparison with Madden Julian Oscillation

The Madden Julian Oscillation (MJO)[29] is a closely related phenomenon. Here, we show that the MJO alone is not sufficient to explain the organization of EREs during JJAS. To do so, we apply the same conditional independence test as for the BSISO using the RMM index as it was introduced by[16]. The qualitative shape of the distribution remains, however, the lower likelihoods express that BSISO1 and BSISO2 indices are more suited for analysis of EREs during boreal summer (Fig. S31).

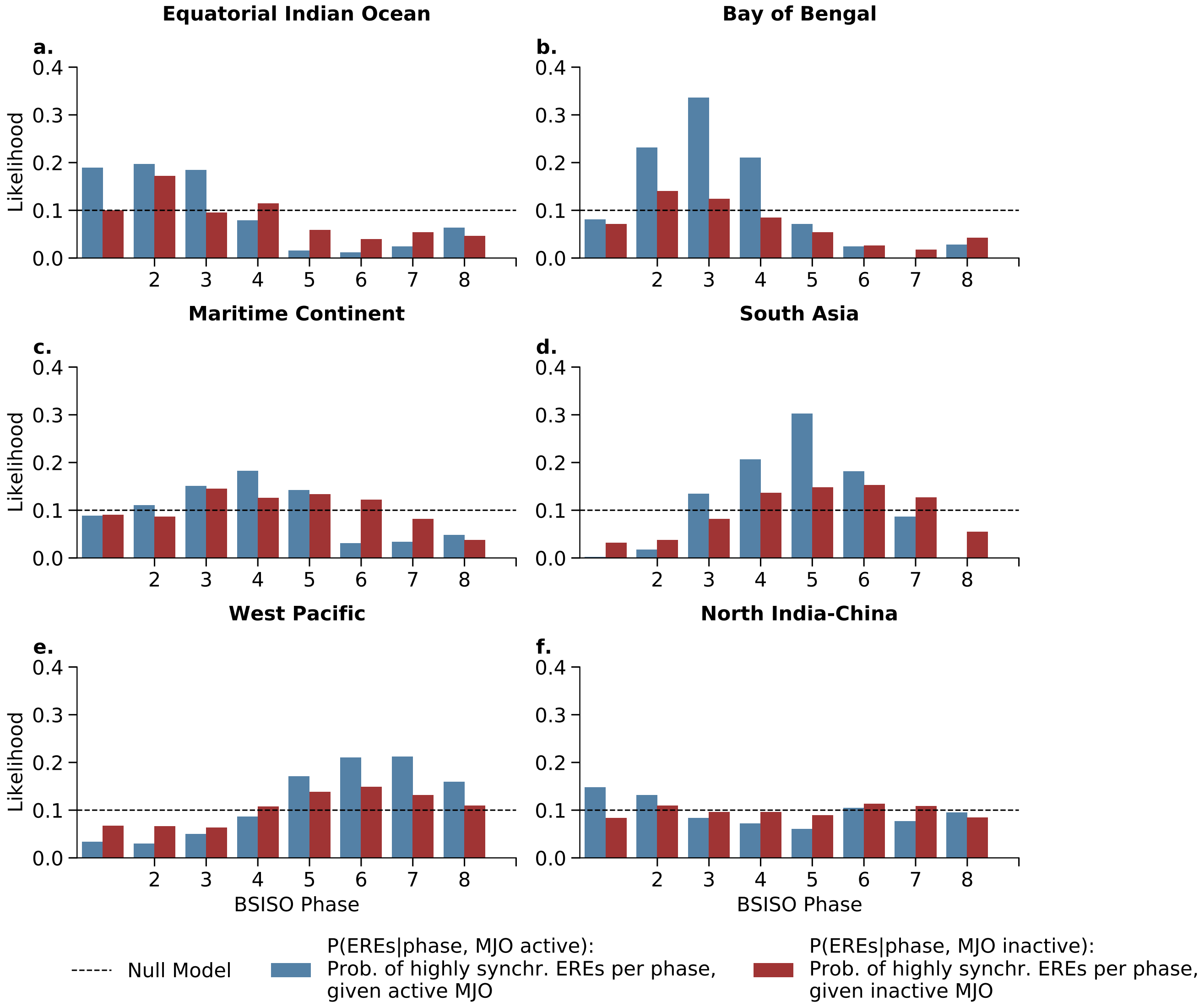

Figure S31. Likelihood of synchronous events for active MJO phases. The occurrence of synchronous events ($s = 1$) is analyzed for active (break) MJO phases displayed as blue (red) bars. The dashed line depicts the likelihood of synchronous events for a threshold of 10 % from a null model of a random distribution of synchronous events. **a-f** Likelihood of synchronous events $s = 1$ for specific phases. The histograms are for the communities received from the analysis in Figure 2 **d**, i.e. **a** Equatorial Indian Ocean, **b** Bay of Bengal, **c** Maritime continent, **d** South Asia, **e** Western Pacific and **f** North India-China.

Supplementary Note 11 Comparison to classical ENSO conditions

The likelihood for a specific propagation mode is substantially increased given the respective ENSO background state (Fig. S32). The conditional dependence test is designed to assess the likelihood of a specific BSISO propagation mode subject to the ENSO state (based on the NINO3.4 index definition[30]). Still, not all samples match exactly with ENSO (Fig. S33). ENSO induces changes in the local zonal as well as the local global meridional overturning circulation. These are visualized in Fig. S34. Also the updrift of vertical winds over the Maritime Continent experiences a modulation by ENSO. This modulation has in turn also influence on the modulation of the BSISO propagation pathways. The differences in the background vertical velocities for the three modes is therefore plotted in Fig.

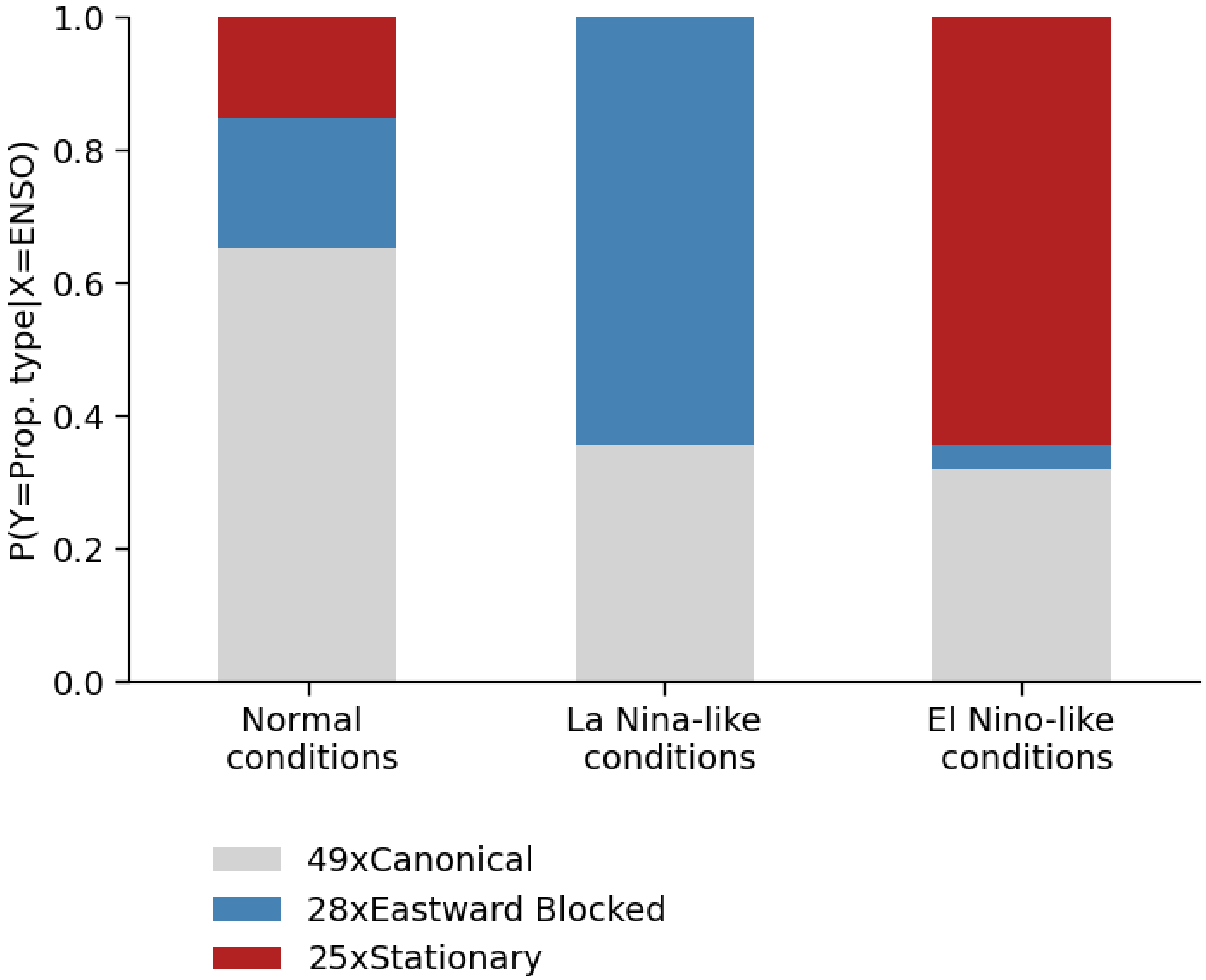

Figure S32. propagation modes at NINO3.4 index. The occurrence of synchronous events is displayed according to the identified propagation mode. The solid line depicts the NINO3.4 index. The definition of an El Niño (La Niña) year was adapted from[31], represented by the dashed line of 0.5 as the respective threshold.

We compare the single BSISO events to the respective ENSO state in the Pacific Ocean. Following[30,32], El Niño and La Niña are defined using the NINO3.4 index. We use June to September daily SST anomalies and select El Niño-like conditions (La Niña-like conditions) based on the average JJAS SST anomalies of the NINO3.4 index region. The respective plot is shown in Fig. S33.

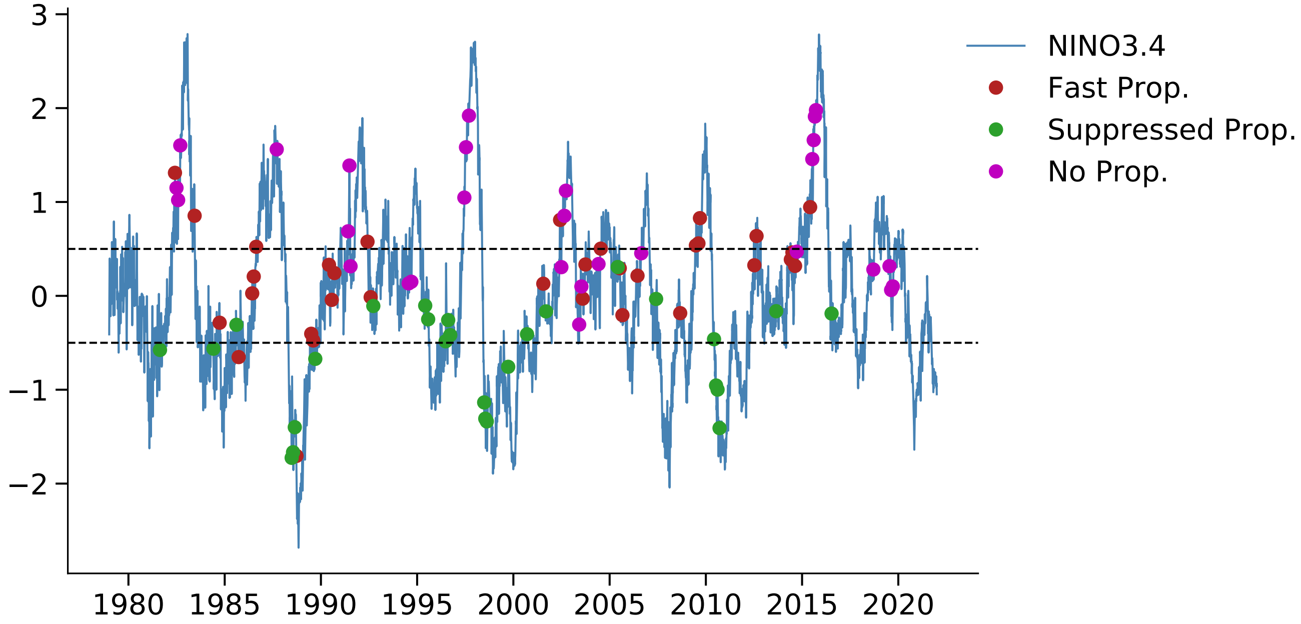

Figure S33. propagation modes at NINO3.4 index. The occurrence of synchronous events is displayed according to the identified propagation mode. The solid line depicts the NINO3.4 index. The definition of an El Niño (La Niña) year was adapted from[32], represented by the dashed line of 0.5 as the respective threshold.

We also analyze the classical overturning circulation conditions for Normal, El Niño-like and La Niña-like conditions in the Pacific Ocean. The pressure level dependent plots are shown in Fig. S34. We further show the spatial structure averaged for 400-600 hPa. The definition of an El Niño, La Niña like condition are based on the NINO3.4 index[32] derived from ERA5 data. The plot is analogous to Fig. 6.

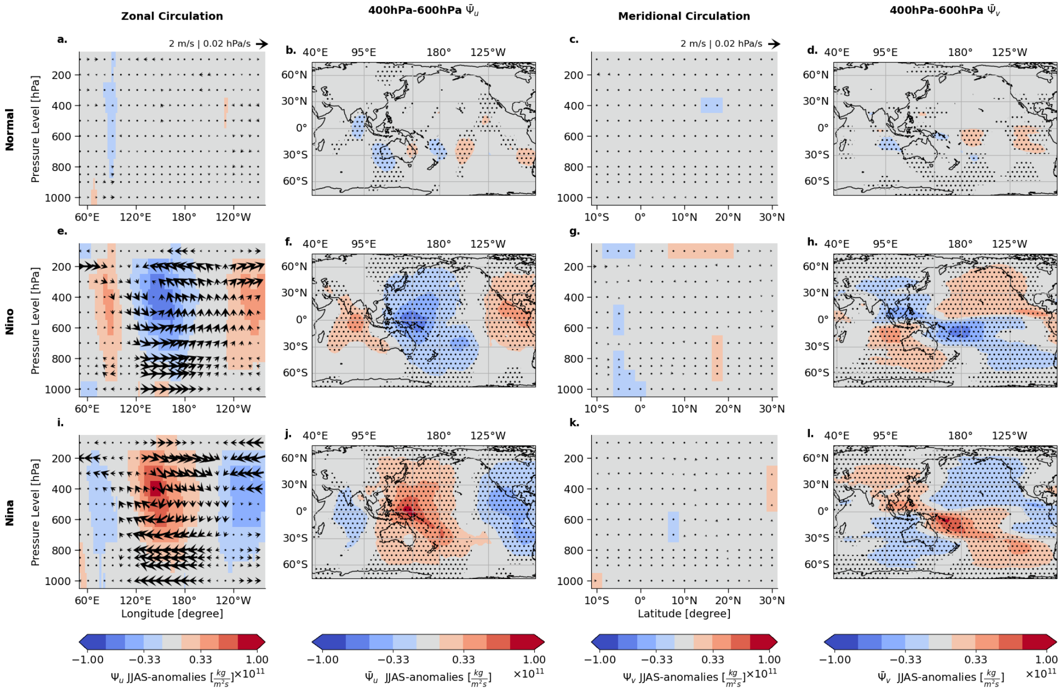

Figure S34. Vertical cuts of overturning circulation for different ENSO types. For the ENSO conditions normal (1st row), El Niño (2nd row) and La Niña (3rd row) the background overturning circulation anomalies are plotted. The ENSO state is based on the NINO3.4 definition[32] and the composited SSTs cover per event the full JJAS period. The first column (**a,c,e**) shows the composited vertical cuts in the zonal direction with the latitudes averaged between 10° S-10° N, the second column (**b,d,f**) in the meridional direction in the Indian Ocean with the latitudes averaged between 60° E-120° E. The color shading denotes the mass stream function in the zonal (meridional) direction. The wind fields are computed in the vertical direction from the u (v) components in m/s and in the horizontal direction in hPa/s.

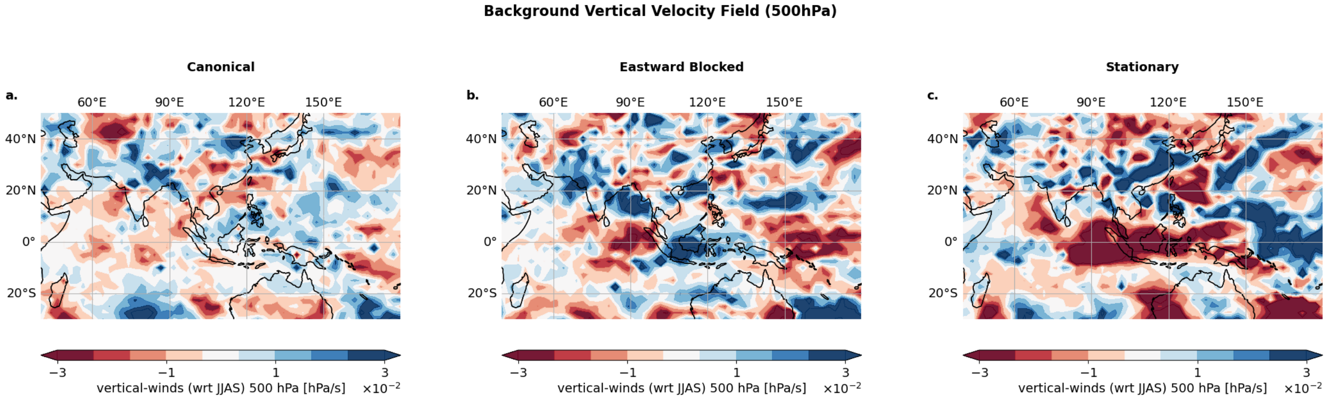

Figure S35. Background Vertical winds at 500 hPa.. The comparison of vertical velocity at 500 hPa is shown for the Canonical (1st column), Eastward Blocked (2nd column), and Stationary mode (3rd column) for the background state, averaged between 15-20 days before the BSISO initiation days. The horizontal direction is measured in hPa/s.

Supplementary Note 12 Characteristics of Moisture mode theory

One recently established mechanism for the canonical north-eastward propagation of the BSISO is the moisture mode theory[33,34]. The BSISO's propagation mechanism is mainly determined by air-sea interaction which is through the feedback processes from updraft air masses associated with BSISO convection. This interaction can be demonstrated by composited plots of downward shortwave radiation (Fig. S36a,d,g), latent heat fluxes (Fig. S36b,e,h), and moisture (Fig. S36c,f,i).

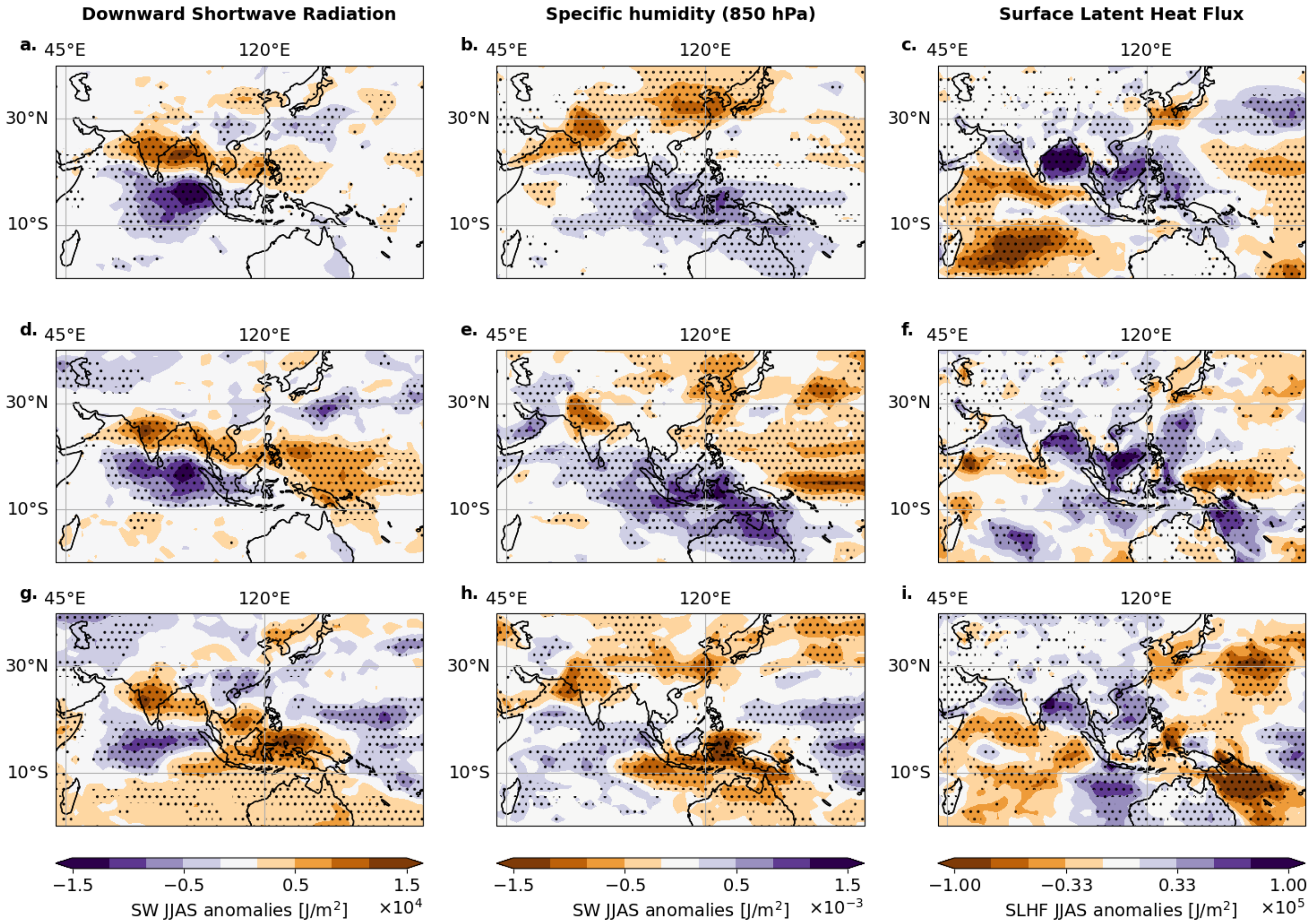

Figure S36. Radiation fluxes analysis. The comparison of Downward Shortwave Radiation (SW), specific humidity (Q) and surface latent heat flux (SLHF) is shown for the Canonical (1st row), Eastward Blocked (2nd row), and Stationary case (3rd row) for the BSISO initiation days. The first column (**a,d,g**) shows the column integrated SW and the second column (**b,e,h**) specific humidity composites. The third column (**c,f,g**) shows the composited surface latent heat flux anomalies. In all subplots, stipples denote anomalies that are significant at a 95 % confidence level using Student's t-test.

Theoretical frameworks[33–35] therefore often use moist static energy (MSE) as the characteristic variable demonstrating the BSISO propagation. Fig. S37a,b,c depict the spatial distribution of rainfall anomalies for the days of maximum synchronization, outgoing longwave radiation and moist static energy (MSE) anomalies at lag 0 day. The positive (negative) anomalies of MJO rainfall align with positive (negative) MSE anomalies, both demonstrating a southeast-northwestward tilt. This agreement

between rainfall and MSE anomalies during the BSISO propagation is in accordance with the moisture mode theory and validates the utilization of MSE analysis to investigate the northward propagation mechanism of the BSISO.

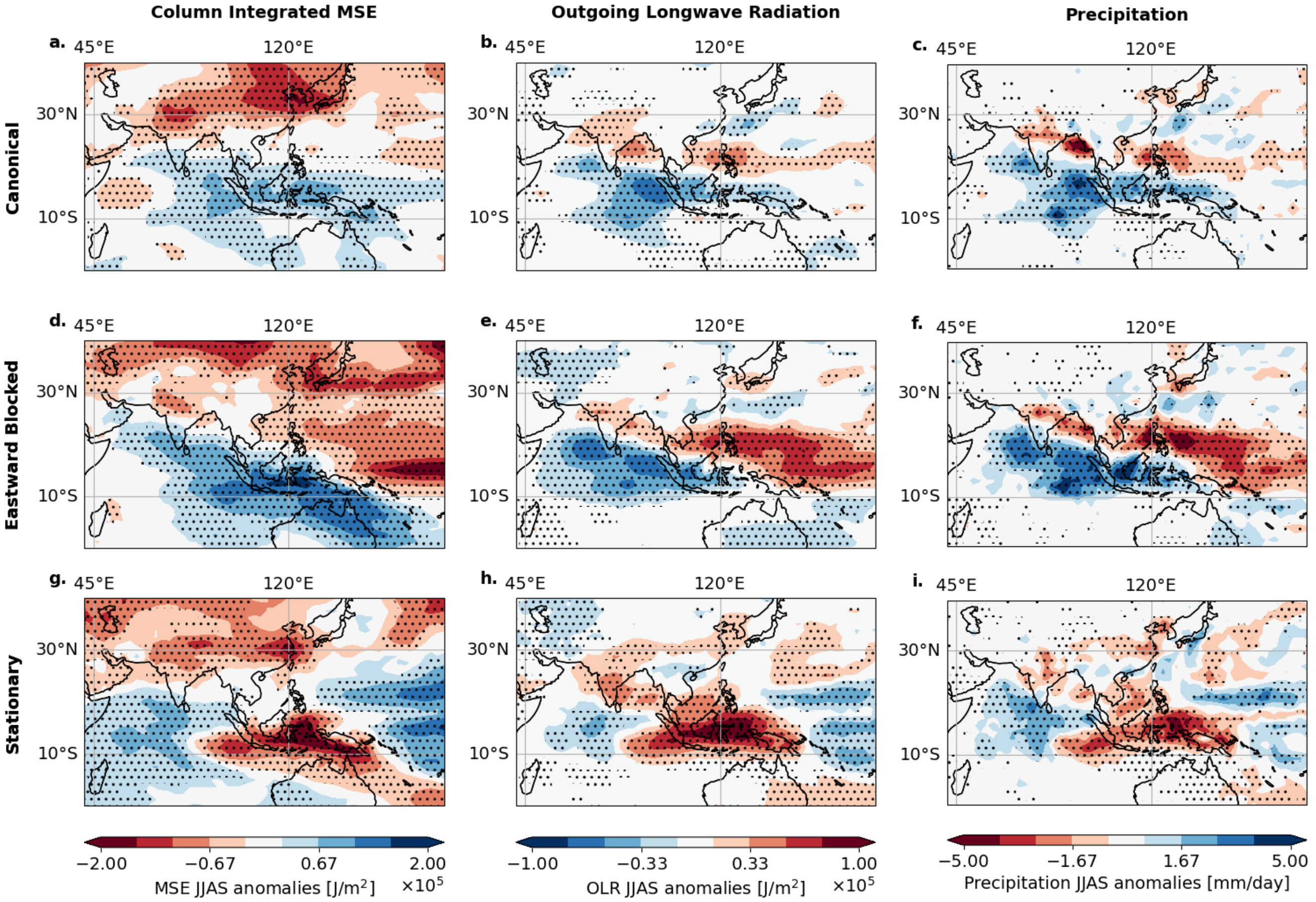

Figure S37. Moisture budget analysis. The comparison of MSE, Outgoing Longwave Radiation (OLR) and precipitation is shown for the Canonical (1st row), Eastward Blocked (2nd row), and Stationary case (3rd row) for the BSISO initiation days. The first column (**a,d,g**) shows the column integrated MSE and the second column (**b,e,h**) OLR composites. The third column (**c,f,g**) shows the composited rainfalls. In all subplots, stipples denote anomalies that are significant at a 95 % confidence level using Student's t-test.

Supplementary Note 13 Vertical Shear Mechanism

One proposed mechanism for the northward propagation of the BSISO is the vertical shear mechanism. It suggests that the easterly negative vertical shear of the zonal mean flow (i.e. low-level westerlies and upper-level easterlies) generates a northward component to the moist Rossby waves[36–38]. As the upward motion w induced by the convective activity in the Indian Ocean decreases northward, the interaction of the BSISO with the easterly vertical shear of the mean flow $\bar{U}_z$ generates cyclonic vorticity ζ_B and boundary layer convergence to the north of the anomalous convection band that promotes the northward movement. This process is described by a two-dimensional barotropic vorticity equation[36]:

$$\frac{\partial \zeta_B}{\partial t} \propto \bar{U}_z \frac{\partial w}{\partial y} \,. \tag{2}$$

Our observed characteristic northward propagation is also in agreement with the vertical shear mechanism[36–38]. The three modes reveal different northward propagation characteristics (Fig. 6 b,d,f) which relate well to the vertical shear mechanism (see Methods). The vertical shear is strongest in the northern Indian Ocean for all three propagation modes (Fig. S38 a,d,g) and hence the necessary condition for northward propagation is fulfilled. The Canonical mode reveals a strong anomalous meridional gradient of the vertical velocity over the Bay of Bengal (Fig. S38 b) and north of the Maritime Continent. This also explains the anomalous relative vorticity pattern over South India and north of the Maritime Continent (Fig. S38 c). We find a similar pattern for the Eastward Blocked mode in the meridional gradients (Fig. S38 e) and thus also in the relative vorticity (Fig. S38 f) with some intensification over the Maritime Continent, which is likely due to the shifted Walker circulation (Fig. 6 f). The Stationary propagation mode is not eastward moving and thus not emitting Rossby Waves upon arriving at the Maritime Continent. Therefore, the region in the northern Indian Ocean does not show significant anomalies in the meridional vertical velocity gradient (Fig. S38 h) and consequently also no significant relative vorticity (Fig. S38 i).

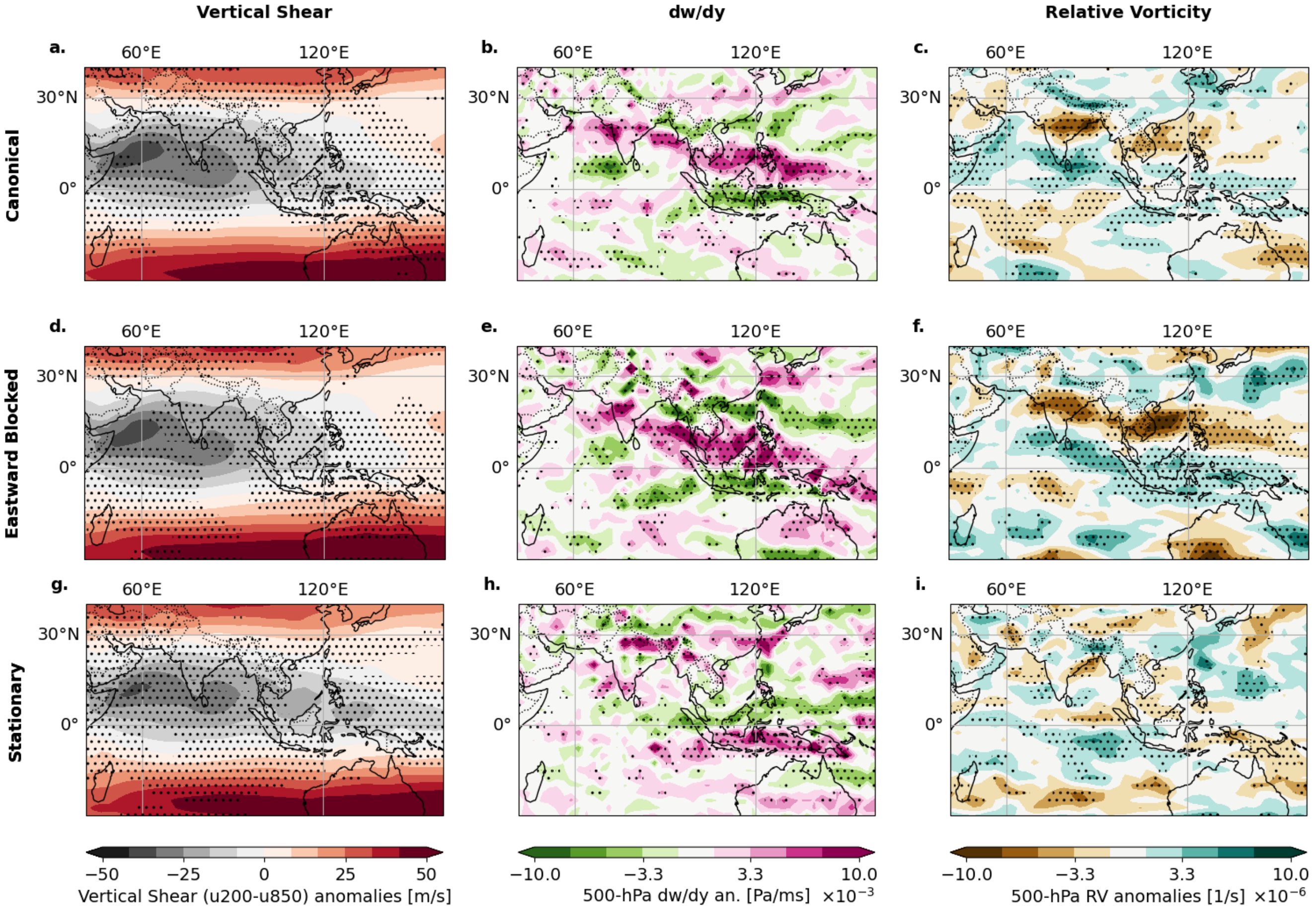

Figure S38. Northward propagation for different propagation modes. The evolution of the northward propagation as a response to eastward propagating Kelvin waves is shown for the Canonical (1st row), Eastward Blocked (2nd row), and Stationary case (3rd row). The first column (**a,d,g**) shows the asymmetrical mean vertical shear $\bar{U}_z$, calculated as $u200 - u850$ and the second column (**b,e,h**) the meridional gradients of the vertical velocity $\frac{\partial w}{\partial y}$ (**c,f,i**). The third column (**c,f,g**) shows the relative vorticity ζ as an indicator of the strength of the northward propagation. All plots are averaged for days 5 to 10 days after initiation. In all subplots, stipples denote anomalies that are significant at a 95 % confidence level using Student's t-test.

Supplementary Note 14 Connection of the rainfall extremes during Indian and East Asian monsoon

One region that emerges from the community detection approach is the NIC community which is of all six regions the only one solely over land (Fig 2 a). The community connects the northeastern part of India, the Tibetan Plateau, the Himalayan Mountains, and most parts of China of which the Himalayan foothills and the Ganges Delta in North East India are among those regions that experience the highest rainfall accumulation during SASM (Fig. S1) during core monsoon season in July.

In early work, the enhanced upper-level atmospheric wave train, known as circumglobal teleconnection[39] (or due to its near-equivalence over Eurasia[40] also known as the "Silk Road pattern"[41]) was suspected to connect the Northern Indian region with the China region[39], and[21] suspect this mechanism to be responsible for the synchronization of EREs between Northern India and Northern China[21]. Composite anomalies of the days of maximum synchronization within the NIC community reveal a large-scale wave train pattern originating from the mid-latitude Atlantic ocean. It is enhanced across Eurasia and connects the Northern India region with the Yellow River basin (Fig. S39) corroborating results from[25].

We also identify a further mechanism connecting parts of Northern India with the Yellow River basin in Northern China. Using composites of vertically integrated moisture vapor flux (IVF) uncovers a continuous path of anomalously high moisture transport established in a moisture corridor (Fig. S40 a-e), starting from the Ganges Delta and gated by the Tibetan Plateau towards the Yellow River Basin and Northern China possibly driven by the Silk Road pattern. The connection manifests itself in the northward displacement of the western North Pacific subtropical high (WNPSH) during the Asian summer monsoon[42] corroborating studies on the dominant route of stage 4 of the East Asian Atmospheric Rivers[42].

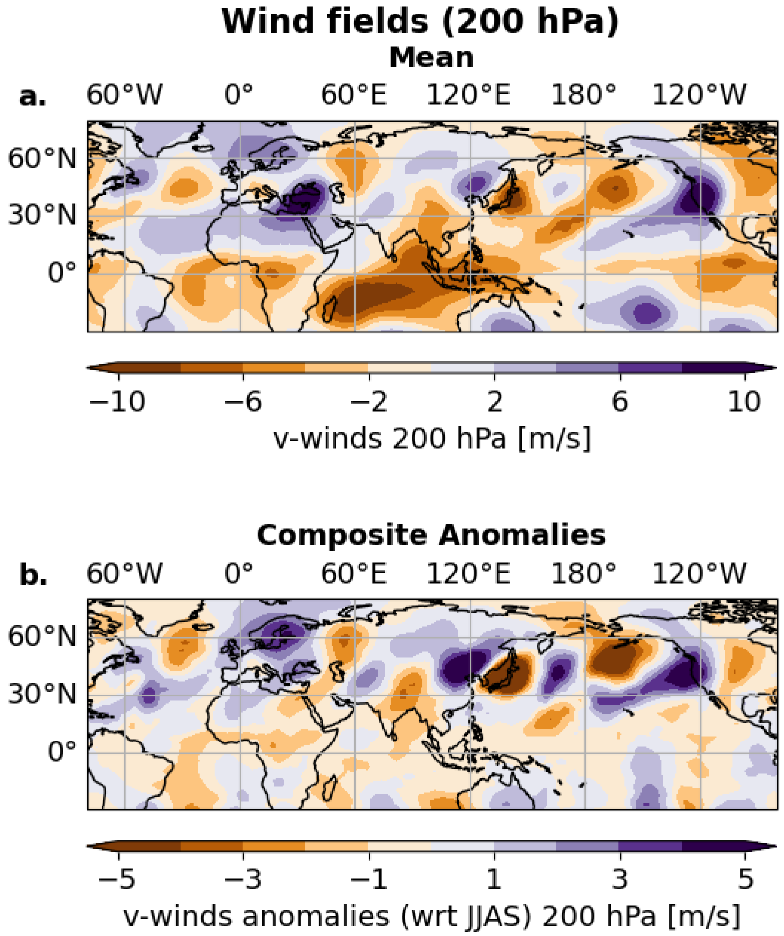

Figure S39. Atmospheric conditions for the synchronous day pattern. The v-winds components at 200 hPa is plotted for the mean (**a**) the composited anomalies **b**). The days were chosen as described in section using the days of maximum synchronization in the NIC region. The composite anomalies are computed with respect to the JJAS climatology. The Silk Road pattern connects clearly the North of India with the China area.

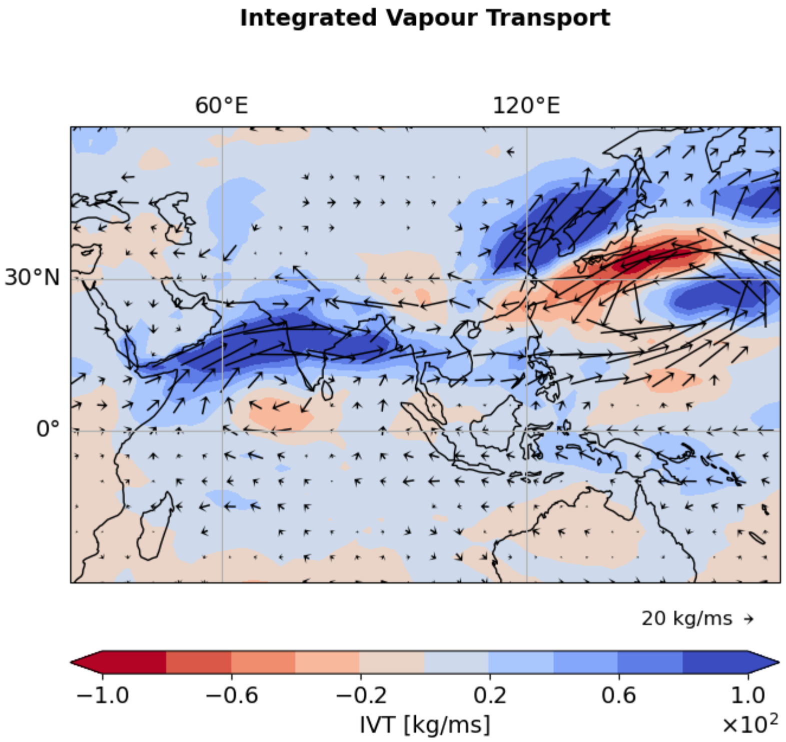

Figure S40. **Vertically integrated water vapor flux (IVT) for days of maximum synchronization for NIC region.** Same as Figure S39 but for the vertically integrated water vapor flux (IVF). Day 0 denotes the days of maximum synchronization. The composite anomalies are computed with respect to the JJAS climatology. Only IVT arrows that are significant at 95 % level following the Student's t-test are plotted.

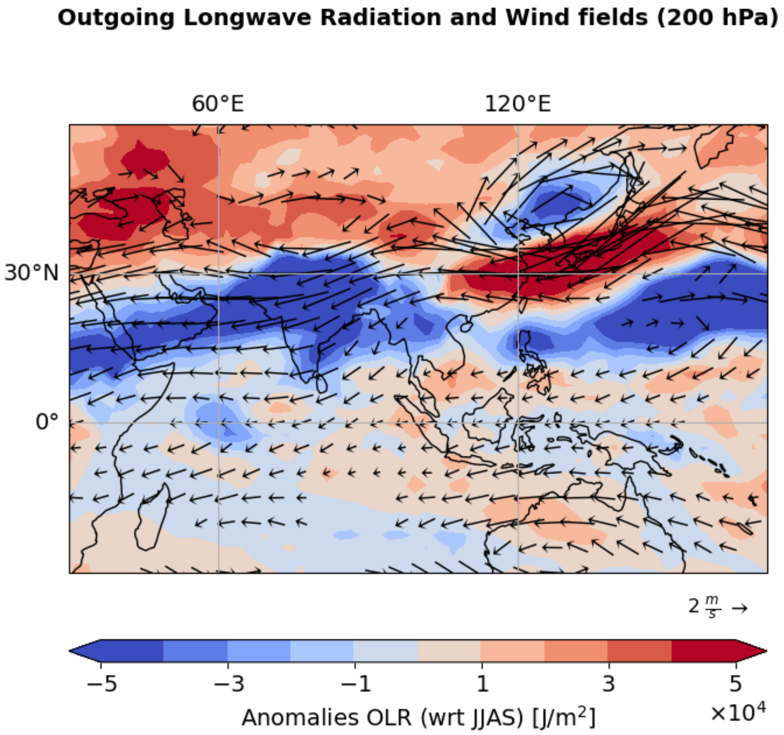

Figure S41. **Outgoing Longwave Radiation for days of maximum synchronization for NIC region** Same as Figure S39 but for Outgoing Longwave Radiation (IVF) overlapped by wind fields at from anomalous upper-level wind fields at 200 hPa. Composites are computed for the days of maximum synchronization. The composite anomalies are computed with respect to the JJAS climatology. Only wind arrows that are significant at 95 % level following the Student's t-test are plotted.

Supplementary References

1. Rheinwalt, A. *et al.* Non-linear time series analysis of precipitation events using regional climate networks for Germany. *Clim. Dyn.* **46**, 1065–1074, DOI: 10.1007/s00382-015-2632-z (2016).

2. Beck, H. E. *et al.* MSWEP V2 Global 3-Hourly 0.1° Precipitation: Methodology and Quantitative Assessment. *Bull. Am. Meteorol. Soc.* **100**, 473–500, DOI: 10.1175/BAMS-D-17-0138.1 (2019).

3. Lau, W. K. M., Waliser, D. E., Majda, A. J. & Stechmann, S. N. Multiscale theories for the MJO. In *Intraseasonal Variability in the Atmosphere-Ocean Climate System*, 549–568, DOI: 10.1007/978-3-642-13914-7_17 (Springer, Berlin, Germany, 2011).

4. Kim, H., Vitart, F. & Waliser, D. E. Prediction of the Madden–Julian Oscillation: A Review. *J. Clim.* **31**, 9425–9443, DOI: 10.1175/JCLI-D-18-0210.1 (2018).

5. Kikuchi, K. The Boreal Summer Intraseasonal Oscillation (BSISO): A Review. *J. Meteorol. Soc. Jpn. Ser. II* 2021–045, DOI: 10.2151/jmsj.2021-045 (2021).

6. Mishra, S. K., Sahany, S. & Salunke, P. Linkages between MJO and summer monsoon rainfall over India and surrounding region. *Meteorol. Atmos. Phys.* **129**, 283–296, DOI: 10.1007/s00703-016-0470-0 (2017).

7. Da Silva, N. A. & Matthews, A. J. Impact of the Madden–Julian Oscillation on extreme precipitation over the western Maritime Continent and Southeast Asia. *Q. J. R. Meteorol. Soc.* **147**, 3434–3453, DOI: 10.1002/qj.4136 (2021).

8. Anandh, P. C., Vissa, N. K. & Broderick, C. Role of MJO in modulating rainfall characteristics observed over India in all seasons utilizing TRMM. *Int. J. Clim.* **38**, 2352–2373, DOI: 10.1002/joc.5339 (2018).

9. Di Capua, G. *et al.* Tropical and mid-latitude teleconnections interacting with the Indian summer monsoon rainfall: a theory-guided causal effect network approach. *Earth Syst. Dyn.* **11**, 17–34, DOI: 10.5194/esd-11-17-2020 (2020).

10. Karmakar, N., Boos, W. R. & Misra, V. Influence of Intraseasonal Variability on the Development of Monsoon Depressions. *Geophys. Res. Lett.* **48**, e2020GL090425, DOI: 10.1029/2020GL090425 (2021).

11. Schreck, C. J. Global Survey of the MJO and Extreme Precipitation. *Geophys. Res. Lett.* **48**, e2021GL094691, DOI: 10.1029/2021GL094691 (2021).

12. Hunt, K. M. R. & Turner, A. G. Nonlinear intensification of monsoon low pressure systems by the BSISO. *Weather. Clim. Dyn. Discuss.* 1–28, DOI: 10.5194/wcd-2022-31 (2022).

13. Li, X., Gollan, G., Greatbatch, R. J. & Lu, R. Impact of the MJO on the interannual variation of the Pacific–Japan mode of the East Asian summer monsoon. *Clim. Dyn.* **52**, 3489–3501, DOI: 10.1007/s00382-018-4328-7 (2019).

14. Kikuchi, K., Wang, B. & Kajikawa, Y. Bimodal representation of the tropical intraseasonal oscillation. *Clim. Dyn.* **38**, 1989–2000, DOI: 10.1007/s00382-011-1159-1 (2012).

15. Wang, S., Ma, D., Sobel, A. H. & Tippett, M. K. Propagation Characteristics of BSISO Indices. *Geophys. Res. Lett.* **45**, 9934–9943, DOI: 10.1029/2018GL078321 (2018).

16. Wheeler, M. C. & Hendon, H. H. An All-Season Real-Time Multivariate MJO Index: Development of an Index for Monitoring and Prediction. *Mon. Weather. Rev.* **132**, 1917–1932, DOI: 10.1175/1520-0493(2004)132<1917:AARMMI>2.0.CO;2 (2004).

17. Lee, J.-Y. *et al.* Real-time multivariate indices for the boreal summer intraseasonal oscillation over the Asian summer monsoon region. *Clim. Dyn.* **40**, 493–509, DOI: 10.1007/s00382-012-1544-4 (2013).

18. Huffman, G. J. *et al.* The TRMM Multisatellite Precipitation Analysis (TMPA): Quasi-global, multiyear, combined-sensor precipitation estimates at fine scales. *J. Hydrometeorol.* **8**, 38–55, DOI: 10.1175/JHM560.1 (2007).

19. Lancichinetti, A. & Fortunato, S. Community detection algorithms: A comparative analysis. *Phys. Rev. E* **80**, 056117, DOI: 10.1103/PhysRevE.80.056117 (2009).

20. Staudt, C. L., Sazonovs, A. & Meyerhenke, H. NetworKit: A Tool Suite for Large-scale Complex Network Analysis. *arXiv* DOI: 10.48550/arXiv.1403.3005 (2014). 1403.3005.

21. Boers, N. *et al.* Complex networks reveal global pattern of extreme-rainfall teleconnections. *Nature* **566**, 373–377, DOI: 10.1038/s41586-018-0872-x (2019).

22. Stolbova, V., Surovyatkina, E., Bookhagen, B. & Kurths, J. Tipping elements of the Indian monsoon: Prediction of onset and withdrawal. *Geophys. Res. Lett.* **43**, 3982–3990, DOI: 10.1002/2016GL068392 (2016).

23. Wolf, F., Ozturk, U., Cheung, K. & Donner, R. V. Spatiotemporal patterns of synchronous heavy rainfall events in East Asia during the Baiu season. *Earth Syst. Dyn.* **12**, 295–312, DOI: 10.5194/esd-12-295-2021 (2021).

24. Malik, N., Marwan, N. & Kurths, J. Spatial structures and directionalities in Monsoonal precipitation over South Asia. *Nonlinear Process. Geophys.* **17**, 371–381, DOI: 10.5194/npg-17-371-2010 (2010).

25. Gupta, S. *et al.* Interconnection between the Indian and the East Asian Summer Monsoon: spatial synchronization patterns of extreme rainfall events. *Int. J. Clim.* **n/a**, DOI: 10.1002/joc.7861 (2022).

26. Gupta, S., Boers, N., Pappenberger, F. & Kurths, J. Complex network approach for detecting tropical cyclones. *Clim. Dyn.* **57**, 3355–3364, DOI: 10.1007/s00382-021-05871-0 (2021).

27. Strnad, F. M., Schlör, J., Fröhlich, C. & Goswami, B. Teleconnection Patterns of Different El Niño Types Revealed by Climate Network Curvature. *Geophys. Res. Lett.* **49**, e2022GL098571, DOI: 10.1029/2022GL098571 (2022).

28. Rheinwalt, A., Marwan, N., Kurths, J., Werner, P. & Gerstengarbe, F.-W. Boundary effects in network measures of spatially embedded networks. *Eur. Lett.* **100**, 28002, DOI: 10.1209/0295-5075/100/28002 (2012).

29. Madden, R. A. & Julian, P. R. Detection of a 40–50 Day Oscillation in the Zonal Wind in the Tropical Pacific. *J. Atmos. Sci.* **28**, 702–708, DOI: 10.1175/1520-0469(1971)028<0702:DOADOI>2.0.CO;2 (1971).

30. Trenberth, K. E. The Definition of El Niño. *Bull. Am. Meteorol. Soc.* **78**, 2771–2778, DOI: 10.1175/1520-0477(1997)078<2771:TDOENO>2.0.CO;2 (1997).

31. Capotondi, A., Wittenberg, A. T., Kug, J.-S., Takahashi, K. & McPhaden, M. J. ENSO Diversity. In *El Niño Southern Oscillation in a Changing Climate*, 65–86, DOI: 10.1002/9781119548164.ch4 (American Geophysical Union (AGU), 2020).

32. Trenberth, K. E. & Stepaniak, D. P. Indices of El Niño Evolution. *J. Clim.* **14**, 1697–1701, DOI: 10.1175/1520-0442(2001)014<1697:LIOENO>2.0.CO;2 (2001).

33. Sobel, A. & Maloney, E. An Idealized Semi-Empirical Framework for Modeling the Madden–Julian Oscillation. *J. Atmos. Sci.* **69**, 1691–1705, DOI: 10.1175/JAS-D-11-0118.1 (2012).

34. Wang, S. & Sobel, A. H. A Unified Moisture Mode Theory for the Madden–Julian Oscillation and the Boreal Summer Intraseasonal Oscillation. *J. Clim.* **35**, 1267–1291, DOI: 10.1175/JCLI-D-21-0361.1 (2022).

35. Adames, Á. F. & Kim, D. The MJO as a Dispersive, Convectively Coupled Moisture Wave: Theory and Observations. *J. Atmos. Sci.* **73**, 913–941, DOI: 10.1175/JAS-D-15-0170.1 (2016).

36. Wang, B. & Xie, X. A Model for the Boreal Summer Intraseasonal Oscillation. *J. Atmos. Sci.* **54**, 72–86, DOI: 10.1175/1520-0469(1997)054<0072:AMFTBS>2.0.CO;2 (1997).

37. Wang, B., Webster, P. J. & Teng, H. Antecedents and self-induction of active-break south Asian monsoon unraveled by satellites. *Geophys. Res. Lett.* **32**, DOI: 10.1029/2004GL020996 (2005).

38. Hoskins, B. & Wang, B. Large-scale atmospheric dynamics. In *The Asian Monsoon*, 357–415, DOI: 10.1007/3-540-37722-0_9 (Springer, Berlin, Germany, 2006).

39. Ding, Q. & Wang, B. Circumglobal Teleconnection in the Northern Hemisphere Summer. *J. Clim.* **18**, 3483–3505, DOI: 10.1175/JCLI3473.1 (2005).

40. Zhou, F., Zhang, R. & Han, J. Relationship between the Circumglobal Teleconnection and Silk Road Pattern over Eurasian continent. *Sci. Bull.* **64**, 374–376, DOI: 10.1016/j.scib.2019.02.014 (2019).

41. Enomoto, T., Hoskins, B. J. & Matsuda, Y. The formation mechanism of the Bonin high in August. *Q. J. R. Meteorol. Soc.* **129**, 157–178, DOI: 10.1256/qj.01.211 (2003).

42. Pan, M. & Lu, M. East Asia Atmospheric River catalog: Annual Cycle, Transition Mechanism, and Precipitation. *Geophys. Res. Lett.* **47**, e2020GL089477, DOI: 10.1029/2020GL089477 (2020).
